# Genome-Wide Identification, Evolutionary and Mutational Analysis of the Buffalo Sox Gene Family

**DOI:** 10.3390/ani13142246

**Published:** 2023-07-08

**Authors:** Muhammad Abdullah, Muhammad Saif-ur Rehman, Muhammad Shah Nawaz-ul Rehman, Abdullah A. AlKahtane, Tahani Mohamed Al-Hazani, Faiz-ul Hassan, Saif ur Rehman

**Affiliations:** 1 Institute of Animal and Dairy Sciences, University of Agriculture, Faisalabad 38040, Pakistanshsaifurrehman@yahoo.com (M.S.-u.R.); 2 Centre for Agricultural Biochemistry and Biotechnology, University of Agriculture, Faisalabad 38040, Pakistan; 3 Department of Zoology, College of Science, King Saud University, Riyadh 11451, Saudi Arabia; 4 Biology Department, College of Science and Humanities, Prince Sattam bin Abdulaziz University, Al-Kharj 11940, Saudi Arabia; t.alhazani@psau.edu.sa; 5 Department of Breeding and Genetics, Cholistan University of Veterinary and Animal Sciences, Bahawalpur 63100, Pakistan; 6 State Key Laboratory for Conservation and Utilization of Subtropical Agro-Bioresources, Guangxi University, Nanning 530005, China

**Keywords:** buffalo, Sox genes, alterations, functional effect, evolution

## Abstract

**Simple Summary:**

The Sox gene family is a set of specific transcriptional factor (TF) proteins, with a very similar sequence compared to the sex-determining region Y (SRY), related high-mobility group (HMG) box genes found in mammals. The Sox gene family is involved in many important developmental processes, including sex determination. In the current study, an *in silico* analysis was performed to provide insights into the evolutionary importance, mutations and gene duplication events of the buffalo Sox gene family. Based on our analysis, we found that the HMG domain was highly conserved throughout the Sox gene family. Mutational analysis revealed twenty non-synonymous mutations with potential detrimental effects on physiological functions in buffalo. The current study concluded that the buffalo Sox gene family was highly conserved throughout evolution, and the non-synonymous mutations identified could potentially be valuable for the selective breeding of buffalo.

**Abstract:**

The Sox gene family constitutes transcription factors with a conserved high mobility group box (HMG) that regulate a variety of developmental processes, including sex differentiation, neural, cartilage, and early embryonic development. In this study, we systematically analyzed and characterized the 20 Sox genes from the whole buffalo genome, using comparative genomic and evolutionary analyses. All the buffalo Sox genes were divided into nine sub-groups, and each gene had a specific number of exons and introns, which contributed to different gene structures. Molecular phylogeny revealed more sequence similarity of buffalo Sox genes with those of cattle. Furthermore, evolutionary analysis revealed that the HMG domain remained conserved in the all members of the Sox gene family. Similarly, all the genes are under strong purifying selection pressure; seven segmental duplications occurred from 9.65 to 21.41 million years ago (MYA), and four potential recombination breakpoints were also predicted. Mutational analysis revealed twenty non-synonymous mutations with potential effects on physiological functions, including embryonic development and cell differentiation in the buffalo. The present study provides insights into the genetic architecture of the Sox gene family in buffalo, highlights the significance of mutations, and provides their potential utility for marker-assisted selection for targeted genetic improvement in buffalo.

## 1. Introduction

The buffalo (*Bubalus bubalis*) is an economically important animal, particularly in Asian countries. It is becoming more popular among agricultural animals as a source of milk, meat, and draft power. Despite its great contribution as a versatile mammal, this species is neglected, as much less research work has been performed, as compared to cattle. Domestic buffalo are divided into two types: the river buffalo and the swamp buffalo. River buffalo are kept for milk and other dairy products, while swamp buffalo are utilized for working in crop fields and meat production [1]. River buffalo are usually found in southwestern Asia, Egypt, India, and southern Europe, whereas swamp buffalo are mainly reared in Southeast Asia, southeast China [2], Thailand, and southern China. Predominantly, buffalo are distributed across India, Pakistan, and China, contributing more than 65% of the global buffalo population.

Understanding the genetic basis of numerous biological processes, such as growth, development, reproduction, and disease susceptibility, is made possible by genomics. Genes underlying specific traits or diseases in animals can be identified to unravel the molecular mechanisms underlying complex traits through functional genomics. Moreover, it can provide deeper insights about the basic tenets of animal biology by investigating the genetic composition of animals [3]. During fetal development, many genes (transcriptional factors) are responsible for the maintenance of the pluripotent state of the embryo, and help in the differentiation of embryonic stem cells [4]. These genes, particularly Sox genes, are involved in sex differentiation, organogenesis, and many other important functional roles in metazoan animals [5].

The Sox gene family expresses a set of specific transcription factor (TF) proteins, with a sequence very similar to the sex-determining region Y (SRY) related high-mobility group (HMG) box genes found in metazoans [6,7]. Several other transcription factors target the major helix of DNA, whereas Sox proteins have a distinctive ability to bind to the minor groove. Sox proteins can interact with other partner proteins in close proximity to DNA as a result of their minor groove binding ability [8]. This ability, combined with the power to bend DNA, has led to the notion that Sox gene encoding proteins can act as architectural proteins, helping to assemble other proteins by organizing chromatin condensation and arranging other DNA-bound transcription factors into physiologically active and structurally defined multiprotein complexes [9]. Based on the conserved section HMG box of the SRY gene (testis determining factor), the presence of the Sox gene family was initially discovered in mammals in 1990 [10]. The Sox gene family has been characterized in a variety of taxa, including vertebrates and invertebrates, owing to the availability of whole-genome sequences in several animals [11,12,13]. Presently, almost 40 Sox genes have been identified in different species, and are classified into nine subgroups. A total of 19 Sox genes have been discovered in chickens [14], 9 in silkworms [15], 20 in mice and humans [16], 8 in common fruit flies [17], 7 in calcareous sponges [6], 14 in starlet sea anemones [18], 11 in sea urchins [19], 27 each in Nile tilapias, zebrafish, and common carp [20,21], and 25 putative Sox genes in channel catfish [12].

Proteins expressed by Sox genes are involved in different biological functions, including sex determination and differentiation, spermatogenesis [5,22,23], organogenesis including neuronal network [24], chondrogenesis [25], and gonadal development and differentiation [26], the development of cartilage [27], the pancreas [28], and eyes [20]. The sequence-specific HMG box domain of the Sox genes, as well as their interconnections with other TFs and cofactors, regulate their activities [15].

Previous research has shown the genome-wide identification of the Sox gene family, phylogenetic analysis, expression analysis, and developmental functions in several metazoan animals [20]. However, no genome wide study of Sox genes in buffalo (*Bubalus bubalis*) is available. The objective of the present study was to identify the members of the Sox gene family and predict their physicochemical properties in buffalo. To evaluate the differences in gene sequences among related species, an *in silico* analysis was performed to provide insights into the evolutionary importance, mutations and gene duplication events of buffalo Sox gene family.

## 2. Materials and Methods

### 2.1. Identification of the Sox Gene Family in Buffalo

Sequences of the Sox gene family of the representative livestock animals including, cattle (*Bos taurus*) having accession numbers (XP_005214070.1, NP_001098933.1, XP_005227617.2, NP_001071596.1, NP_001076940.1, NP_001178347.1, XP_024851815.1, XP_002698019.3, XP_024836864.1, NP_001180176.1, XP_024855327.1, XP_010809935.2, XP_005216751.1, NP_001157253.1, NP_001179004.1, NP_001193180.1, NP_001069257.1, XP_024855919.1, NP_001039894.1, ABY19364.1), sheep (*Ovis aries*) (XP_027829705.1, NP_001305003.1, XP_027819161.1, XP_027815027.1, XP_042103325.1, XP_027835157.1, XP_027820413.2, XP_027817142.1, XP_027829812.2, XP_027823898.1, XP_042101688.1, XP_027832844.1, XP_027831988.1, XP_011958232.1, XP_004012716.2, XP_027828760.1, XP_027832715.1, XP_042110920.1, XP_027826507.2, CAA82946.1), goat (*Capra hircus*) (XP_017911809.1, NP_001272601.1, XP_017899578.1, XP_017894532.1, XP_005680807.1, XP_017915078.1, ENSCHIT00000022852.1, XP_017896058.1, XP_017919394.1, XP_017904232.1, XP_017911193.1, XP_017913158.1, XP_017915490.1, XP_005675585.2, XP_005693578.2, XP_017913995.1, XP_017912969.1, XP_017911941.1, XP_017907206.1, AXF84123.1), and buffalo (*Bubalus bubalis*) (XP_025118363.1, XP_006056297.2, XP_025132502.3, XP_025121975.3, XP_045021534.1, XP_045018884.1, XP_006064379.2, XP_025130606.3, XP_025135172.2, XP_006071428.1, XP_025117398.3, XP_025119497.1, XP_025133526.2, XP_025147052.1, XP_006062923.4, XP_025121191.1, XP_025119695.1, XP_025118466.2, XP_006058618.4, AAR02416.1) were downloaded from online genome databases NCBI (https://www.ncbi.nlm.nih.gov/ (accessed on 22 September 2022), and Ensemble (http://asia.ensembl.org/index.html (accessed on 24 September 2022). To determine putative Sox genes in these livestock species, the Hidden Markov model (HMM) profile of the conserved HMG box domain protein (DNA binding protein) sequence from the Pfam database [29] was used to search for their predicted protein-coding variants, using a local BLASTP tool with an E values set at 10^−^^5^. Using the TBLASTN tool, the genomic pattern of Sox genes from these livestock species was further determined by comparing the query protein sequence with all six possible reading frames of the database.

### 2.2. Multiple Sequence Alignment and Phylogenetic Analysis of Buffalo Sox Genes

All Sox gene sequences were aligned using ClustalW with the default parameters, and the alignments were manually adjusted [30]. A phylogenetic tree of closely related species was constructed using MEGA 11 [31] by implementing neighbor joining (NJ) method and setting a bootstrap of 1000 replicates. The results of multiple sequence alignments and phylogeny were visualized and examined using iTOL v4.2.3 [32].

### 2.3. Gene Structure, Motif Pattern, and Conserved Domain Analysis

The buffalo Sox genes were mapped to their respective chromosomes by determining their chromosomal positions, as indicated by the buffalo whole genome sequence (Table S1). The nucleotide sequences associated with each identified Sox gene were obtained from the NCBI and Ensemble databases. Based on the alignments of their coding sequences and related genomic sequences, the exon–intron patterns of the Sox genes were identified. Multiple expectation maximization for motif elicitation (MEME) [33] and the conserved domain database were used [34] to identify the motif pattern, and to analyze the evolutionary conserved domain of the Sox gene family in buffalo. The minimum and maximum width of motifs were set to 6 and 50, respectively, and the number of motifs was set at 10. Then, using in-house scripts in the TBtools program, the final gene structure was presented and analyzed using the buffalo genome annotation file in the general feature format (GFF) [35].

### 2.4. Prediction of Physicochemical Properties

Physiochemical properties of Sox proteins, including protein size, molecular weight, instability index, aliphatic index, amino acid number, and isoelectric points, were determined using ExPASy [36]. The hydrophobicity and hydrophilicity of protein peptides were also calculated using the grand average of hydropathicity index (GRAVY) in the PROTPRAM tool [37].

### 2.5. Comparative Amino Acid Analysis

The amino acid sequences of all Sox genes were compared among buffalo, cattle, goat, and sheep using the ClustalW. Mutations, including insertions and deletions in amino acid sequences of Sox genes, were identified using the PROMPT tool [38]. Amino acid substitutions in Sox gene protein sequences of different livestock species were evaluated using the multiple align show (http://www.bioinformatics.org/SMS/multi_align.html (accessed on 10 September 2022)) tool. Several online tools, including (Sorting Intolerant from Tolerant) SIFT [39], Provean [40], PolyPhen 2 (Polymorphism Phenotyping v2) [41], I-Mutant [42], phdSNP [43], MuPro [44], SNAP [45], predict SNP [46], and meta-SNP [47] were used to investigate the potential effects of identified variations on protein structure and functions.

### 2.6. Synteny Analysis and Chromosome Localization

Using genomic resources, the chromosomal localization of buffalo Sox genes was obtained. The genome annotation file was used as an input in MCScanX to map the actual locations of Sox genes on chromosomes [48]. Tbtools was used to generate a dual synteny plot to identify collinear Sox genes between buffalo and cattle. In addition, the frequency of gene duplication for the buffalo Sox gene family was evaluated by a pairwise alignment of homologous gene pairs of Sox genes, using MEGA11 v.11.0 and the MUSCLE program [31]. Furthermore, the ratio of nonsynonymous (Ka) to synonymous (Ks) nucleotide substitution rates was calculated to measure selective pressure, and pairwise combinations of genes were also determined using the ka/ks calculation tool (http://services.cbu.uib.no/tools/kaks) (assessed on 17 January 2023).

### 2.7. Selection Analysis of the Sox Gene Family

To measure the site-specific purifying and positive selection on the buffalo Sox gene family, the Selection server (http://selecton.tau.ac.il/ (accessed on 20 January 2023)), which applies a Bayesian inference strategy for evolutionary models, was used. The coding sequences (in FASTA format) of all buffalo Sox genes were analyzed in the SELECTION server [49] to detect evolutionary forces at single amino acid sites. The M8 evolutionary model, which allows positive selection to act on the protein, was implemented in this investigation. A fraction p0 of sites are selected from a beta distribution (specified in the interval 0), and a fraction p1(=1 − p0) of the sites are selected from an additional category ωs (specified as ≥1). Thus, sites selected from the beta distribution are those undergoing purifying selection, whereas sites selected from the ωs category are those undergoing neutral or positive selection [49].

### 2.8. Analysis of Recombination Breakpoints in the Sox Gene Family

To detect recombination breakpoints in multiple sequence aligned buffalo Sox genes, the Genetic Algorithm Recombination Detection (GARD) tool was used [50]. The approach is designed to look for evidence of segment-specific phylogenies from the beginning. The strategy looked for B or fewer breakpoints in the alignment if the maximum number of breakpoints (B, which can also be inferred) is known. It also inferred phylogenies for each prospective non-recombinant segment, and evaluated the goodness of a match-up using a relevant data criterion, such as the small subset Akaike information criterion (AIC) [51], which was derived from a maximum likelihood model fit with each component.

## 3. Results

### 3.1. Genome-Wide Identification of Buffalo Sox Genes

**Figure 1:**
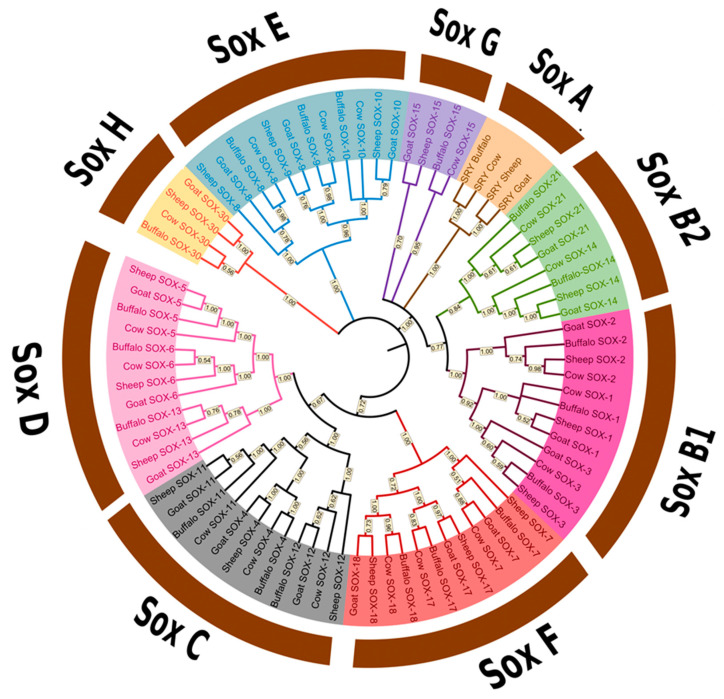
Phylogenetic tree of Sox gene family in buffalo and other related species (including sheep, goat, and cattle) constructed using neighbor joining method with a bootstrap value of 1000.

Using the BLAST and HMMER tools, a total of 20 Sox genes were discovered in the buffalo genome, and classified into nine subgroups. A total of 80 non-redundant protein sequences encoded by 20 Sox genes were predicted from the whole genomes of four mammalian species, viz. goat, sheep, cattle, and buffalo (Figure 1). All representative species have the same number of Sox genes in their genome.

### 3.2. Phylogenetic Analysis of the Sox Gene Family

A phylogenetic analysis of Sox genes from four mammalian species was performed, and all identified genes were classified into nine primary subgroups. The SoxA subgroup contains the SRY gene; the SoxB2 subgroup contains Sox14 and Sox21 genes; SoxB1 subgroup contains Sox1, Sox2, and Sox3 genes; the SoxF subgroup contains Sox7, Sox17, and Sox18 genes; the SoxC subgroup contains Sox4, Sox11, and Sox12 genes; the SoxD subgroup contains Sox5, Sox6, and Sox13 genes; and the SoxG subgroup contains Sox15 gene. Most of the Sox genes in the buffalo Sox gene family showed more homology with cattle than with goats or sheep. The resulting dendrogram (Figure 1) also showed that the buffalo Sox gene family was closely related to other representative mammalian species. The buffalo SRY gene shared sequence homology with cattle, while the goat SRY gene was more similar to sheep. Similarly, the Sox4, Sox9, Sox15, Sox17, Sox18, and Sox30 genes share similar sequence homology pattern in representative species.

### 3.3. Gene Structure Characterization of the Buffalo Sox Gene Family

**Figure 2:**
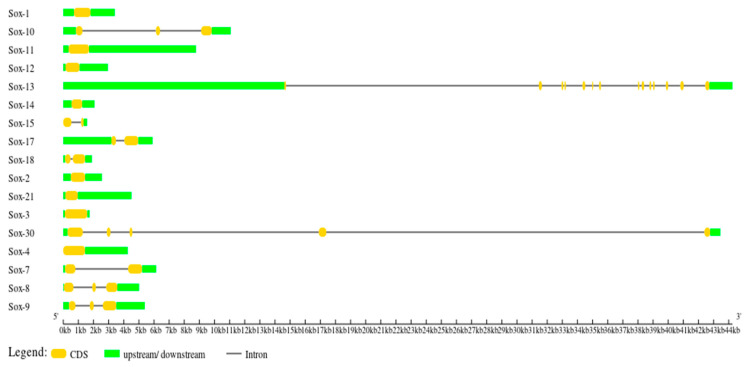
Gene structures of buffalo Sox gene family.

**Figure 3:**
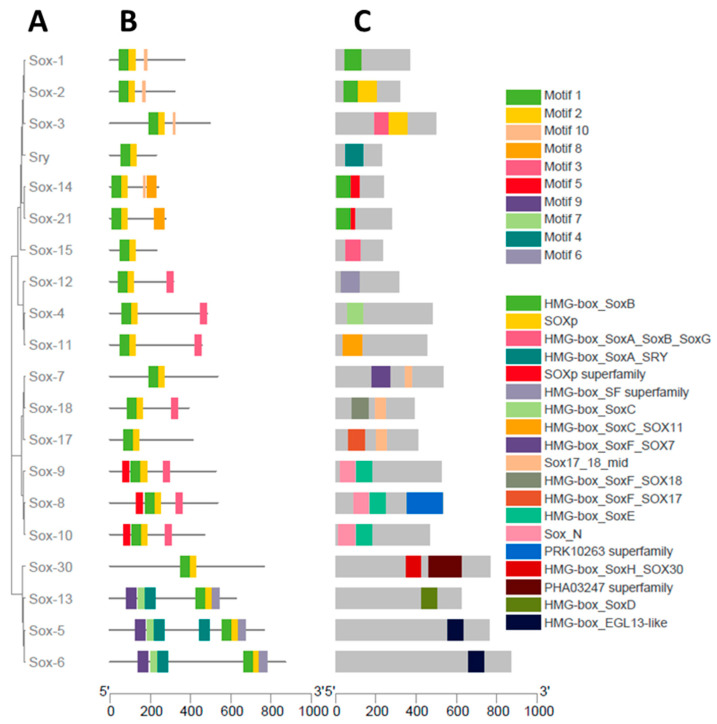
Buffalo Sox gene family. (**A**) Phylogenetic relationship, (**B**) motif orientation, and (**C**) conserved domain distribution.

The structural features of buffalo Sox genes, including gene structure (Figure 2), phylogenetic relationship (Figure 3A), motif orientation (Figure 3B), and conserved domain distribution (Figure 3C), were analyzed. These analyses were performed with respect to the information obtained from the phylogenetic tree. The gene structure analysis revealed that the introns and UTRs structures differed substantially, and each buffalo Sox gene had a specific number of exons and introns (Figure 2). Furthermore, conserved domain analysis illustrated the presence of the HMG domain in all Sox genes, whereas the SoxN domain was found only in the SoxE group. Furthermore, a total of 10 MEME conserved motifs were identified from buffalo Sox genes, of which three motifs (MEME-1, MEME-4, and MEME-9) had a higher number of amino acids (Table 1). Motif 1 was annotated as HMG domain, and motif 4 was annotated as SoxN, after the Pfam search (Table 1). The peptide length in the Sox gene family ranged from 229 (SRY) to 870 (Sox6) amino acids (Table 2).

### 3.4. Physiochemical Attributes of the Buffalo Sox Gene Family

The physicochemical properties of the buffalo Sox gene family were investigated, including its chromosomal distribution, exon number, amino acid length, molecular weight (Da), isoelectric point (pI), instability index (II), aliphatic index (AI), and grand average of hydropathicity index (GRAVY), as shown in Table 2. The molecular weight of the buffalo Sox gene family ranged from 25,180.29 to 96,885.84 (MW), and the isoelectric point (pI) value ranged from 4.95 and 9.90. All the Sox proteins behave as basic, except Sox5, Sox9, Sox10, Sox11, Sox12, Sox13, and Sox17, which exhibit acidic properties (Table 2). Only four Sox proteins (Sox5, Sox13, Sox21, and Sox30) were thermostable, with aliphatic index values greater than 65. According to the instability index values, which were higher than 40, all members of the Sox gene family were highly unstable. Based on lower GRAVY values, all buffalo Sox proteins were hydrophilic (Table 2).

### 3.5. Comparative Amino Acid Analysis of the Buffalo Sox Gene Family

All amino acid sequences of the buffalo Sox gene family were compared with cattle to check the amino acid variation. Five buffalo Sox genes, including Sox2, Sox3, Sox10, Sox12, and Sox14, had no amino acid substitutions, while eight buffalo Sox genes, including Sox1, Sox2, Sox3, Sox4, So6, Sox7, Sox9, and Sox18, had indels. Single insertions were observed at the 39, 27, 400, 287, 147, 388, and 298 positions of Sox1, Sox2, Sox4, Sox6, Sox7, Sox9, and Sox18 genes, respectively, while two insertions were observed in the Sox3 gene, one of which one was a 45 amino acid insertion and the other one was a single amino acid (Table 3).

Furthermore, a total of 80 amino acid substitutions were detected in buffalo Sox genes, where one single amino acid variation was found in the Sox1, Sox4, Sox11, and Sox21 genes, two variations were found in the Sox5, Sox9, Sox17, and Sox18 genes, four in Sox, and Sox15, and five and six amino acid substitutions were found in the Sox6 and Sox8 genes, respectively. Likewise, 11 mutations were detected in the Sox13 gene, 17 mutations in Sox30, and a total of 21 mutations were found in buffalo SRY gene (Figures S1–S20). Furthermore, the functional effect of these mutations was evaluated using different software, and a total of 20 amino acid substitutions were identified in different Sox genes of buffalo, including Sox5 (P362S), Sox6 (L2P), Sox8 (Y443F), Sox13 (G5R, F20V, and F39L), Sox15 (T205P), Sox30 (P28A, C53W, T179R, Y605F, and E696D), and SRY (V63L, W64G, R69K, R70Q, D38K, W92G, A112S, and Q172H), which showed non-synonymous mutations that were expected to have an overall detrimental effect on protein structure and functions (Table S1). In contrast, the other amino acid substitutions had an overall synonymous effect, with no impact on protein structure and function.

### 3.6. Synteny Analysis and Chromosome Localization of the Sox Gene Family in Buffalo

**Figure 4:**
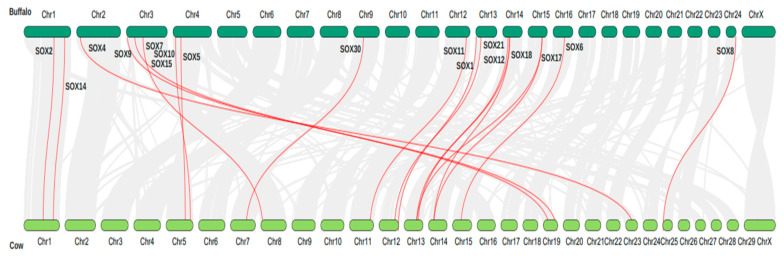
Collinearity analysis of Sox genes family in buffalo and cattle.

In buffalo, all of the Sox gene isoforms were distributed on 11 chromosomes, while in cattle, they are present on 12 chromosomes. Additionally, in buffalo, most of the Sox genes were found near the terminal ends of the chromosomes (Figure 4). To better understand the evolutionary history, the gene duplication events of the buffalo Sox gene family were examined. The homologous gene pairs Sox17/Sox18, Sox8/Sox9, Sox30/Sox13, Sox5/Sox6, Sox21/Sox7, Sox11/Sox4, and Sox3/Sox1 were all found to be duplicated, and were considered to be segmental duplications (Table 4).

Additionally, for these homologous gene pairs, the ratio of nonsynonymous substitutions per nonsynonymous site (Ka) to synonymous substitutions per synonymous site (Ks) was determined (Table 4). All seven gene pairs were under purifying selection pressure, with a Ka/Ks ratio <1. Similarly, four gene pairs (Sox17/Sox18, Sox11/Sox4, Sox3/Sox1 and Sox21/Sox7) were duplicated in the Tortonian age, between 7.246 to 11.63 mya (Table 4), after one gene pair (Sox8/Sox9) was duplicated in the Serravallian. In addition, two gene pairs (Sox5/Sox6 and Sox30/Sox13) were duplicated in Aquitanian stage, around 21 mya ago (Table 4).

### 3.7. Single Amino Acid Site Evolution

**Figure 5:**
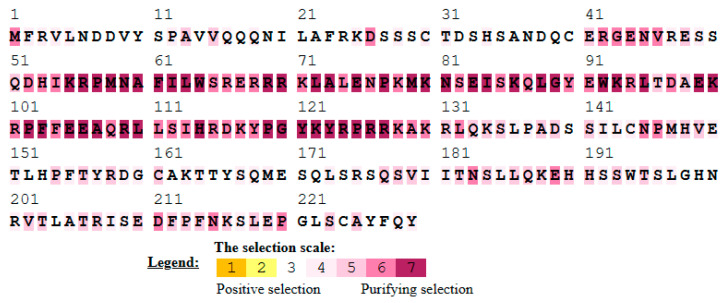
Site-specific evolutionary forces on the Sox gene family members.

To analyze the site-wise selection of the buffalo Sox gene family, we evaluated a buffalo Sox gene data set using SELECTION server to determine the Ka and Ks ratio at each amino acid site. As shown in Figure 5, the Sox gene family is under purifying selection pressure. In our data set, we found out that 40–130 amino acids in the sequence were under strong purifying selection, as indicated by their ka/ks ratio.

### 3.8. Recombination Analysis Using Genetic Algorithm Recombination Detection (GARD)

**Figure 6:**
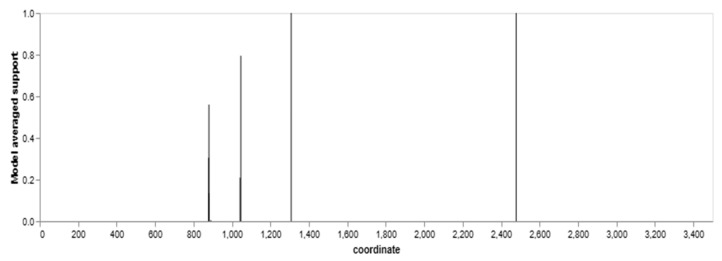
Model-averaged support for breakpoint placement.

GARD found evidence of recombination breakpoints by examining the 10,492 models at a rate of 5.71 models per second. The alignment contained 2165 potential breakpoints, translating into a search space of 395,463,521,942,023 models with up to 5 breakpoints, of which 0.00% was explored by the genetic algorithm (Figure S21). The model-averaged support for the breakpoint sites was calculated by quantifying the model-averaged frequency of seeing a breakpoint at a particular site across all points in the alignment. It was based on the standardized Akaike weights of the models, for which the plots were congruent with the best-fitting model. By performing multiple breakpoint analyses using a genetic algorithm, the multiple sequence alignment of 20 Sox gene nucleotide sequences with 3117 sites, of which 2166 were variable, revealed four major recombination breakpoints at sites 880, 1046, 1308, and 2478 (Figure 6).

**Figure 7:**
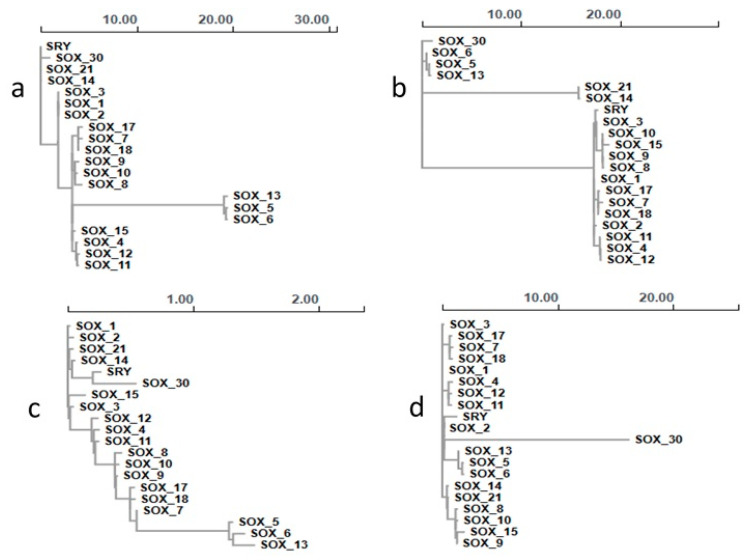
Trees for individual fragments. (**a**) Tree 1, coordinate range 1–880, (**b**) tree 2, coordinate range 881–1046, (**c**) tree 3, coordinate range 1047–1308, and (**d**) tree 4, coordinate range 1309–2478.

Analyses of fragmented sequences indicated by GARD-discovered recombination breakpoints revealed phylogenetic segregation (Figure 7) across the different recombination fragments trees in the coordinate ranges 1–880 (Figure 7a), 881–1046 (Figure 7b), 1047–1348 (Figure 7c), and 1349–2046 (Figure 7d).

## 4. Discussion

Recent developments in high-throughput genome sequencing technologies, such as next-generation sequencing, have made it easier for researchers to find SNPs in the genome of an organism, and detect synonymous or non-synonymous mutations that may have a potential impact on the individual [52,53,54]. These SNPs have the potential to cause structural or functional abnormalities in the translated gene’s final product [55,56]. High-throughput sequencing technologies can help researchers to better understand the genetics of animals at a molecular level. For mammals, the study of candidate genes requires all available genetic resources to discover and identify those genes that have functional roles, like production ability, disease resistance, adaptation, and productivity, and also to study their interaction with other genes [57]. Comparative genomic analysis has helped researchers explore the genetics behind commercially important functional traits, by uncovering novel genes and their regulatory pathways that could have a significant impact on the progress of the buffalo industry [54].

In the present study, characterization of the Sox gene family in the buffalo genome revealed 20 Sox genes. Phylogenetic analysis indicated that most of the Sox genes had more similarity between “buffalo and cattle”, and “sheep and goat” and were grouped into 9 subgroups. Previously, the analysis of Sox gene family revealed 19 members of Sox genes in chickens [14], 20 in mice and humans [16], and 27 in common carp [21]. Structural analysis of the buffalo Sox gene family was performed, and the top ten conserved motifs were analyzed, in which motif 1 and motif 5 were annotated as HMG and Sox-N domain after searching out in the Pfam database. Previously, it was shown that HMG (high mobility group) domain is a 70 amino acid long, highly conserved sequence in mammals, found in Sox gene family members [58].

The physicochemical properties of the buffalo Sox proteins exhibited identical properties in the same group, which is consistent with earlier research [59]. In vitro stability of proteins is indicated by an instability index (II) value of less than 40 [60]. In this study, all members of the Sox gene family were found to be highly unstable, based on higher instability index values. The high instability of all the Sox gene family members observed in this study may have significant implications for their biological function and potential applications in selective breeding. Aliphatic index values of higher than 65 for the proteins show a thermostable property [61]. Only Sox5, Sox13, Sox21, and Sox30 proteins exhibited thermostable properties in the present study. The GRAVY values for all Sox protein family members were found to be negative in the current study, indicating their hydrophilic nature. A negative GRAVY value indicates that the protein is hydrophilic in nature and has a higher solubility in aqueous solutions, with its subcellular location likely to be within the nucleus, indicating that it is not a membrane protein [62,63]. This information may have important implications for the function and stability of Sox proteins, which could be explored in future studies.

To acquire new genes or genetic polymorphisms, organisms employ gene duplication systems, such as retroposition, genome or chromosome duplication, and crossing over, which are essential for the evolution of physiological mechanisms [64]. Although the frequency of duplication is difficult to quantify, selective pressure and mutations with functional consequences are essential for the development of duplicated genetic variations [65]. In the present study, seven segmental duplication events were observed in the Sox gene family. The Ka/Ks ratio also showed that all duplicated gene pairs were found under purifying selection pressure (Ka/Ks ratio < 1) [66]. The results from the SELECTION server also justify that there is a strong purifying selection on the Sox gene family in the 40–130 amino acid region in buffalo, indicating that this is a highly conserved region. This strong purifying selection in the Sox gene family may have potential benefits in selective breeding for the improvement of desirable traits. It is important to note that a strong overall purifying selection may mask multiple codon sites under positive selection [67,68]. About 70% (14 out of 20) of the Sox genes were duplicated in the present study, indicating an important expansion of Sox gene family members in buffalo.

An exciting area of research in recent years has been the identification and measurement of evolutionary forces that have led to genetic diversity [69]. These techniques are now widely employed in the statistical toolkit for sequence analysis [70]. In this study, a recombination analysis using GARD revealed that there are four potential breakpoints in the nucleotide sequences of Sox genes. These breakpoints are the reason why the Sox gene family has diverse functions in different species. Strong evolutionary forces impose variations in the sequences, and this can lead to variable functions [50]. Comparing the AIC scores of the best-fitting GARD model (62,144.6), which allows for different topologies between segments, and the model (6515.7), which assumes the same tree for all the partitions inferred by GARD but allows for different branch lengths between partitions, leads to the conclusion that at least one of the breakpoints does indeed reflect a topological incongruence. It would be interesting to explore the reason for the topological incongruence, as it may be related to a biological or evolutionary process that created it, or the characteristics of the species tree [71]. Moreover, it should be examined in a methodological context, such as the extent to which error or uncertainty in phylogenetic inference affects the perception of topological incongruence between gene trees and the species tree [72].

Comparative genomics is a vast, integrated strategy that compares and investigates the biology of individual genomes to identify similarities and variations across genomes of different species [73,74,75]. Several members of the Sox gene family have been conserved between cattle and buffalo, including Sox2, Sox3, Sox10, Sox12, and Sox14. Comparative genomic studies of cattle and buffalo have shown up to 97% homology [76,77]. This high level of genomic homology indicates that cattle and buffalo are close on the evolutionary scale, so that cheap and fast solutions can be realized to transfer the latest genomic technologies between the species. Moreover, homology between these two species is important for understanding the variations in physiological traits and possible convergent evolutions of these species [54,78]. Mutations can be beneficial, by increasing overall fitness, or might be detrimental, by affecting the structural and functional properties of translated products [79,80]. By comparing the buffalo genome with the cattle genome, single amino acid substitutions in Sox genes were identified. Previously, researchers revealed 21 SNPs in the SRY coding sequence of Brahman crossbred cattle (produced by crossing with Belgian Blue and Wagyu bulls), in which they found 71% non-synonymous and 29% synonymous variations [81]. The transversion mutation at position T1707G changes the amino acid from phenylalanine to cystine [81].

In the present study, comparative analyses of the coding sequence of SRY gene between buffalo and other related mammals have shown the conservation of sequences in these species. The SRY gene is an important member of the Sox gene family, which is mainly involved in sex determination and testis differentiation [5,10,23,82]. Due to its susceptibility to recombination with the X chromosome in the meiotic XY pair, this gene is one of the most-conserved Y-specific genes during evolution in a variety of mammals [83]. Considering this property, researchers have used this gene as a molecular maker to find out the patrilineal phylogeny of Bovidae and other similar species [84]. The SRY genes of Chinese native cattle and yaks were cloned and sequenced, and the results showed less divergence of the coding region of SRY gene among these species [85]. In the present study, 20 amino acid changes with non-synonymous effects were observed between cattle and buffalo SRY coding regions, of which nine were present in the HMG region and eleven changes were detected outside HMG region, which is consistent with the previous investigation [86]. Further studies are warranted to reveal the physiological manifestation of these changes in the coding region of the SRY gene. Obviously, these variations in the buffalo SRY coding region have potential benefits in understanding the evolutionary relationship between cattle and buffalo, identifying functional differences, developing molecular markers, and improving animal productivity [54,78]. The SRY gene has shown copy number variations (CNVs) only in Vietnamese and Laotian buffalo, indicating its evolutionary significance. Moreover, the SRY gene is mainly associated with sex differentiation and male sexual development, and can potentially be used as a candidate marker for sperm quality and fertility in bulls, due to CNVs observed within and across species [87].

In the current study, the mutational effects were observed, which indicated eight non-synonymous mutations in the SRY gene. Previously, mutations in different regions of the SRY gene were reported in patients of partial or complete gonadal dysgenesis [88,89,90,91,92,93,94,95,96,97]. Furthermore, other studies also highlighted the critical role of the Sox8 gene in human reproduction. They found that male infertility and a variety of abnormalities, including 46, XY DSD (disorder in sex development) and 46, XX POI (primary ovarian insufficiency), are caused by mutations in the Sox8 gene [98]. Similarly, it was also reported that patients with mental retardation or growth impairment with significant language disorder, behavioral issues, and mild dysmorphic traits have been shown to have Sox5 mutations. These findings suggest that Sox5 mutations may affect neuronal activity and/or development [99,100,101]. Thus, it can be inferred from previous investigations that the mutations found in the present study might have a significant functional role in sex differentiation and evolutionary processes. The single amino acid substitutions detected in the present study can also be used as a marker to detect genetic diversity between buffalo and other related species [102].

## 5. Conclusions

The present study identified 20 Sox genes from the buffalo genome. Molecular phylogenetic analysis revealed high structural and sequence similarity between cattle and buffalo Sox genes. Furthermore, evolutionary analysis revealed that the HMG domain is conserved in all members of Sox gene family. Similarly, all the genes are under strong purifying selection pressure, and seven segmental duplications that occurred from 9.65 to 21.41 MYA, as well as four potential recombination breakpoints, were also predicted. Mutational analysis revealed twenty non-synonymous mutations with potential detrimental effects on buffalo physiology. The current study concluded that the buffalo Sox gene family is highly conserved throughout evolution, and the non-synonymous mutations identified could potentially be valuable for the selective breeding of buffalo. However, further research is needed to determine the potential physiological consequences of the identified mutations in buffalo.

## Figures and Tables

**Table 1:** Differentially conserved motifs in buffalo Sox gene family.

Motif	Protein Sequence	Length (No. of Amino Acids)	Pfam Domain
MEME-1	IKRPMNAFMVWAKDZRRKLAQZNPDMHNAEISKRLGKEWKLLSESEKRPF	50	HMG
MEME-2	IEEAERLRAQHMKDYPDYKYRPRRKKKTL	29	-
MEME-3	HFDFPDYDTPELSEEIAGBWETFDVAELDFYL	32	-
MEME-4	QKKLAASQIEKQRQQMELARQQQEQIARQQQQLLQQQHKINLLQQQIQQV	50	-
MEME-5	DDKFPVCIREAVSQVLKGYDWTLVPMPVRVNG	32	SoxN
MEME-6	VDGKKLRIGEYKALMRSRRQEMRQYFTVGQQPQIPIAT	38	-
MEME-7	EIKGTPESLAEKERQLLVMINQLTSLREQLLAAHDE	36	-
MEME-8	HTHSHPSPGNPGYMIPCNCSAWPAPGLQPPLAYILFPGMGKPGJDPY	47	-
MEME-9	VTFGTPERRKGSLADVVDTLKQKKLEELIKNEPEESPCIEKLLSKDWKEK	50	-
MEME-10	HWEQPVYTTLTR	12	-

**Table 2:** Physicochemical properties of the Sox gene family in *Bubalus bubalis*.

Sr. No.	Gene	Chr	Exon	AA	MW	pI	II	AI	(GRAVY)
1	Sox1	13	1	370	37,471.04	9.7	51.56	56.51	−0.462
2	Sox2	1	1	321	34,538.03	9.74	59.66	48.1	−0.738
3	Sox3	X	1	497	49,942.45	9.9	71.26	60.42	−0.371
4	Sox4	2	2	482	47,842.16	7.2	58.7	54.32	−0.473
5	Sox5	4	27	764	84,193.73	6.15	63.43	67.98	−0.758
6	Sox6	16	27	870	96,885.84	7.08	62.81	63.4	−0.839
7	Sox7	3	2	534	57,021.97	9.53	62.66	55.47	−0.822
8	Sox8	24	3	534	56,084.84	7.42	59.58	52.62	−0.687
9	Sox9	3	3	525	57,358.68	6.31	81.38	46.7	−0.999
10	Sox10	4	4	469	50,020.2	6.19	58.52	53.3	−0.822
11	Sox11	12	1	455	47,465.32	4.95	66.32	57.65	−0.653
12	Sox12	14	1	314	33,997.76	5.14	67.74	49.87	−0.982
13	Sox13	5	17	666	73,923.77	6.35	70.28	71.37	−0.729
14	Sox14	1	1	240	26,485.39	9.68	53.51	63.17	−0.585
15	Sox15	3	2	233	25,180.29	9.85	71.44	50.73	−0.855
16	Sox17	15	2	410	43,036.69	5.91	65.3	60.39	−0.512
17	Sox18	14	2	391	41,533.25	8.42	75.69	63.12	−0.569
18	Sox21	13	1	277	28,696.95	9.74	57.93	68.77	−0.217
19	Sox30	9	5	766	83,895.1	8.92	67.12	70.08	−0.61
20	SRY	Y	1	229	26,425.82	9.47	63.01	63.49	−0.884

Note. [Sr. No., serial number; Chr, chromosome; AA, amino acid number; MW, molecular weight; II, instability index; pI, isoelectric point; AI, aliphatic index; GRAVY, grand average of hydropathicity index].

**Table 3:** Identification of indels in buffalo Sox gene family compared to cattle.

Gene	Indel	Position	Amino Acid/s
Sox1	Insertion	39	G
Sox2	Insertion	27	G
Sox3	Insertion	1–45	MIGQGASLQACQSPGLRVARGGPSPNPEGSEQVYKRPGERPTRLR
Sox3	Insertion	188	G
Sox4	Insertion	400	S
Sox6	Insertion	287	Q
Sox7	Insertion	1–147	MRGWSPAPAPGPRDHRRLPPPGRRHLRCELAGRGAAPGLRGTDPREPPGRRRGGPGAGARWGSGPPPASPPGRSERGRCGPGRRGARAVKEGGAAPPSRVIGGRSLSKLINKGPGRGCRPSWTPQPVRGPGQRRPDDAKRGDPRAAM
Sox9	Insertion	388	Q
Sox18	Insertion	298–299	GP

**Table 4:** Analysis of the Ka/Ks ratio and evolutionary time scale for each duplicated gene pair of the buffalo Sox genes.

Gene Pairs	Chromosome	Duplication	Ka	Ks	Ka/Ks	Time (MYA)
Sox17/Sox18	15/14	SD	0.12	0.21	0.56	9.65
Sox8/Sox9	24/3	SD	0.16	0.28	0.58	12.66
Sox30/Sox13	9/5	SD	0.40	0.47	0.85	21.41
Sox5/Sox6	16/4	SD	0.11	0.46	0.25	21.07
Sox21/Sox7	13/3	SD	0.18	0.22	0.81	10.17
Sox11/Sox4	12/2	SD	0.12	0.21	0.56	9.65
Sox3/Sox1	X/13	SD	0.09	0.22	0.39	10.32

Note: According to the geological time scale, the Tortonian and Serravallian are two stages/ages of the Neogene period, which occurred approximately 23 to 2.6 million years ago. The Tortonian age is estimated to have spanned from approximately 11.6 to 7.2 million years ago, while the Serravallian age is estimated to have spanned from approximately 13.8 to 11.6 million years ago.

## Data Availability

All data generated in this study is presented in the manuscript.

## Supplementary Materials

The following supporting information can be downloaded at: https://www.mdpi.com/article/10.3390/ani13142246/s1, Figure S1: Comparative amino acid analyses of Sox1 genes in cattle, buffalo, sheep, and goats; Figure S2: Comparative amino acid analyses of Sox2 genes in cattle, buffalo, sheep, and goats; Figure S3: Comparative amino acid analyses of Sox3 genes in cattle, buffalo, sheep, and goats; Figure S4: Comparative amino acid analyses of Sox4 genes in cattle, buffalo, sheep, and goats; Figure S5: Comparative amino acid analyses of Sox5 genes in cattle, buffalo, sheep, and goats; Figure S6: Comparative amino acid analyses of Sox6 genes in cattle, buffalo, sheep, and goats; Figure S7: Comparative amino acid analyses of Sox7 genes in cattle, buffalo, sheep, and goats; Figure S8: Comparative amino acid analyses of Sox8 genes in cattle, buffalo, sheep, and goats; Figure S9: Comparative amino acid analyses of Sox9 genes in cattle, buffalo, sheep, and goats; Figure S10: Comparative amino acid analyses of Sox10 genes in cattle, buffalo, sheep, and goats; Figure S11: Comparative amino acid analyses of Sox11 genes in cattle, buffalo, sheep, and goats; Figure S12: Comparative amino acid analyses of Sox12 genes in cattle, buffalo, sheep, and goats; Figure S13: Comparative amino acid analyses of Sox3 genes in cattle, buffalo, sheep, and goats; Figure S14: Comparative amino acid analyses of Sox3 genes in cattle, buffalo, sheep, and goats; Figure S15: Comparative amino acid analyses of Sox15 genes in cattle, buffalo, sheep, and goats; Figure S16 Comparative amino acid analyses of Sox17 genes in cattle, buffalo, sheep, and goats; Figure S17: Comparative amino acid analyses of Sox18 genes in cattle, buffalo, sheep, and goats; Figure S18: Comparative amino acid analyses of Sox21 genes in cattle, buffalo, sheep, and goats; Figure S19: Comparative amino acid analyses of Sox30 genes in cattle, buffalo, sheep, and goats; Figure S20: Comparative amino acid analyses of SRY genes in cattle, buffalo, sheep, and goats; Figure S21: Analysis of GARD; Table S1: Buffalo and cattle Sox gene family with chromosome number and location; Table S2: Functional effects of mutations in Sox genes in buffalo.

## References

[1] Sanghuayphrai N., Nakavisut S., Dongpaletum C., Phothikanit G., Supanun S. (2013). Genetic Parameters and Trends for Weaning Weight and Calving Interval of Department of Livestock Development Swamp Buffalo. Editor. Board.

[2] Lu X., Duan A., Liang S., Ma X., Deng T. (2020). Genomic Identification, Evolution, and Expression Analysis of Collagen Genes Family in Water Buffalo during Lactation. Genes.

[3] Rexroad C., Vallet J., Matukumalli L.K., Reecy J., Bickhart D., Blackburn H., Boggess M., Cheng H., Clutter A., Cockett N. (2019). Genome to Phenome: Improving Animal Health, Production, and Well-Being–a New USDA Blueprint for Animal Genome Research 2018–2027. Front. Genet..

[4] Prasad C.S., Palanisamy A., Satheshkumar S., Gomathy V.S., Raj G.D., Thangavel A. (2013). Sox-2 Gene Expression Pattern in Stem Cells Derived from Different Stages of in Vitro Produced Buffalo (*Bubalus Bubalis*) Embryos. Buffalo Bull..

[5] Jiang T., Hou C.-C., She Z.-Y., Yang W.-X. (2013). The SOX Gene Family: Function and Regulation in Testis Determination and Male Fertility Maintenance. Mol. Biol. Rep..

[6] Fortunato S., Adamski M., Bergum B., Guder C., Jordal S., Leininger S., Zwafink C., Rapp H.T., Adamska M. (2012). Genome-Wide Analysis of the Sox Family in the Calcareous Sponge Sycon Ciliatum: Multiple Genes with Unique Expression Patterns. Evodevo.

[7] Yu J., Zhang L., Li Y., Li R., Zhang M., Li W., Xie X., Wang S., Hu X., Bao Z. (2017). Genome-Wide Identification and Expression Profiling of the SOX Gene Family in a Bivalve Mollusc Patinopecten Yessoensis. Gene.

[8] van de Wetering M., Oosterwegel M., van Norren K., Clevers H. (1993). Sox-4, an Sry-like HMG Box Protein, Is a Transcriptional Activator in Lymphocytes. EMBO J..

[9] Werner M.H., Burley S.K. (1997). Architectural Transcription Factors: Proteins That-Remodel DNA. Cell.

[10] Gubbay J., Collignon J., Koopman P., Capel B., Economou A., Münsterberg A., Vivian N., Goodfellow P., Lovell-Badge R. (1990). A Gene Mapping to the Sex-Determining Region of the Mouse Y Chromosome Is a Member of a Novel Family of Embryonically Expressed Genes. Nature.

[11] Koopman P., Schepers G., Brenner S., Venkatesh B. (2004). Origin and Diversity of the SOX Transcription Factor Gene Family: Genome-Wide Analysis in Fugu Rubripes. Gene.

[12] Zhang S., Chen X., Wang M., Zhang W., Pan J., Qin Q., Zhong L., Shao J., Sun M., Jiang H. (2018). Genome-Wide Identification, Phylogeny and Expressional Profile of the Sox Gene Family in Channel Catfish (*Ictalurus Punctatus*). Comp. Biochem. Physiol. Part D Genom. Proteom..

[13] Yao C., Wan H., Zhang Z., Lin J., Wang Y. (2020). Genome-Wide Identification and Expression Profile of the Sox Gene Family in Different Tissues and during Embryogenesis in the Pacific White Shrimp (*Litopenaeus Vannamei*). Gene.

[14] Takada S., Mano H., Koopman P. (2005). Regulation of Amh during Sex Determination in Chickens: Sox Gene Expression in Male and Female Gonads. Cell. Mol. Life Sci. C.

[15] Wei L., Cheng D., Li D., Meng M., Peng L., Tang L., Pan M., Xiang Z., Xia Q., Lu C. (2011). Identification and Characterization of Sox Genes in the Silkworm, Bombyx Mori. Mol. Biol. Rep..

[16] Schepers G.E., Teasdale R.D., Koopman P. (2002). Twenty Pairs of Sox: Extent, Homology, and Nomenclature of the Mouse and Human Sox Transcription Factor Gene Families. Dev. Cell.

[17] Crémazy F., Berta P., Girard F. (2001). Genome-Wide Analysis of Sox Genes in Drosophila Melanogaster. Mech. Dev..

[18] Magie C.R., Pang K., Martindale M.Q. (2005). Genomic Inventory and Expression of Sox and Fox Genes in the Cnidarian Nematostella Vectensis. Dev. Genes Evol..

[19] Howard-Ashby M., Materna S.C., Brown C.T., Chen L., Cameron R.A., Davidson E.H. (2006). Gene Families Encoding Transcription Factors Expressed in Early Development of Strongylocentrotus Purpuratus. Dev. Biol..

[20] Wei L., Yang C., Tao W., Wang D. (2016). Genome-Wide Identification and Transcriptome-Based Expression Profiling of the Sox Gene Family in the Nile Tilapia (*Oreochromis niloticus*). Int. J. Mol. Sci..

[21] Zafar I., Iftikhar R., Ahmad S.U., Rather M.A. (2021). Genome Wide Identification, Phylogeny, and Synteny Analysis of Sox Gene Family in Common Carp (*Cyprinus Carpio*). Biotechnol. Rep..

[22] Kent J., Wheatley S.C., Andrews J.E., Sinclair A.H., Koopman P. (1996). A Male-Specific Role for SOX9 in Vertebrate Sex Determination. Development.

[23] Kozhukhar V.G. (2012). SRY and SOX9: The Main Genetic Factors of Mammalian Sex Determination. Tsitologiia.

[24] Sandberg M., Källström M., Muhr J. (2005). Sox21 Promotes the Progression of Vertebrate Neurogenesis. Nat. Neurosci..

[25] Wright E., Hargrave M.R., Christiansen J., Cooper L., Kun J., Evans T., Gangadharan U., Greenfield A., Koopman P. (1995). The Sry-Related Gene Sox9 Is Expressed during Chondrogenesis in Mouse Embryos. Nat. Genet..

[26] Janssen R., Andersson E., Betnér E., Bijl S., Fowler W., Höök L., Leyhr J., Mannelqvist A., Panara V., Smith K. (2018). Embryonic Expression Patterns and Phylogenetic Analysis of Panarthropod Sox Genes: Insight into Nervous System Development, Segmentation and Gonadogenesis. BMC Evol. Biol..

[27] Suzuki T., Sakai D., Osumi N., Wada H., Wakamatsu Y. (2006). Sox Genes Regulate Type 2 Collagen Expression in Avian Neural Crest Cells. Dev. Growth Differ..

[28] McDonald E., Krishnamurthy M., Goodyer C.G., Wang R. (2009). The Emerging Role of SOX Transcription Factors in Pancreatic Endocrine Cell Development and Function. Stem Cells Dev..

[29] El-Gebali S., Mistry J., Bateman A., Eddy S.R., Luciani A., Potter S.C., Qureshi M., Richardson L.J., Salazar G.A., Smart A. (2019). The Pfam Protein Families Database in 2019. Nucleic Acids Res..

[30] Thompson J.D., Gibson T.J., Higgins D.G. (2003). Multiple Sequence Alignment Using ClustalW and ClustalX. Curr. Protoc. Bioinform..

[31] Tamura K., Stecher G., Kumar S. (2021). MEGA11: Molecular Evolutionary Genetics Analysis Version 11. Mol. Biol. Evol..

[32] Letunic I., Bork P. (2021). Interactive Tree Of Life (ITOL) v5: An Online Tool for Phylogenetic Tree Display and Annotation. Nucleic Acids Res..

[33] Bailey T.L., Boden M., Buske F.A., Frith M., Grant C.E., Clementi L., Ren J., Li W.W., Noble W.S. (2009). MEME SUITE: Tools for Motif Discovery and Searching. Nucleic Acids Res..

[34] Marchler-Bauer A., Anderson J.B., DeWeese-Scott C., Fedorova N.D., Geer L.Y., He S., Hurwitz D.I., Jackson J.D., Jacobs A.R., Lanczycki C.J. (2003). CDD: A Curated Entrez Database of Conserved Domain Alignments. Nucleic Acids Res..

[35] Chen C., Chen H., Zhang Y., Thomas H.R., Frank M.H., He Y., Xia R. (2020). TBtools: An Integrative Toolkit Developed for Interactive Analyses of Big Biological Data. Mol. Plant.

[36] Artimo P., Jonnalagedda M., Arnold K., Baratin D., Csardi G., De Castro E., Duvaud S., Flegel V., Fortier A., Gasteiger E. (2012). ExPASy: SIB Bioinformatics Resource Portal. Nucleic Acids Res..

[37] Gasteiger E., Hoogland C., Gattiker A., Wilkins M.R., Appel R.D., Bairoch A. (2005). Protein Identification and Analysis Tools on the ExPASy Server. Proteom. Protoc. Handb..

[38] Schmidt T., Frishman D. (2006). PROMPT: A Protein Mapping and Comparison Tool. BMC Bioinform..

[39] Sim N.-L., Kumar P., Hu J., Henikoff S., Schneider G., Ng P.C. (2012). SIFT Web Server: Predicting Effects of Amino Acid Substitutions on Proteins. Nucleic Acids Res..

[40] Choi Y., Chan A.P. (2015). PROVEAN Web Server: A Tool to Predict the Functional Effect of Amino Acid Substitutions and Indels. Bioinformatics.

[41] Adzhubei I., Jordan D.M., Sunyaev S.R. (2013). Predicting Functional Effect of Human Missense Mutations Using PolyPhen-2. Curr. Protoc. Hum. Genet..

[42] Doss C.G.P., Rajith B., Garwasis N., Mathew P.R., Raju A.S., Apoorva K., William D., Sadhana N.R., Himani T., Dike I.P. (2012). Screening of Mutations Affecting Protein Stability and Dynamics of FGFR1—A Simulation Analysis. Appl. Transl. Genom..

[43] Capriotti E., Fariselli P. (2017). PhD-SNPg: A Webserver and Lightweight Tool for Scoring Single Nucleotide Variants. Nucleic Acids Res..

[44] Khan S., Vihinen M. (2010). Performance of Protein Stability Predictors. Hum. Mutat..

[45] Bromberg Y., Rost B. (2007). SNAP: Predict Effect of Non-Synonymous Polymorphisms on Function. Nucleic Acids Res..

[46] Bendl J., Musil M., Štourač J., Zendulka J., Damborský J., Brezovský J. (2016). PredictSNP2: A Unified Platform for Accurately Evaluating SNP Effects by Exploiting the Different Characteristics of Variants in Distinct Genomic Regions. PLoS Comput. Biol..

[47] Capriotti E., Altman R.B., Bromberg Y. (2013). Collective Judgment Predicts Disease-Associated Single Nucleotide Variants. BMC Genom..

[48] Wang Y., Tang H., DeBarry J.D., Tan X., Li J., Wang X., Lee T., Jin H., Marler B., Guo H. (2012). MCScanX: A Toolkit for Detection and Evolutionary Analysis of Gene Synteny and Collinearity. Nucleic Acids Res..

[49] Doron-Faigenboim A., Stern A., Mayrose I., Bacharach E., Pupko T. (2005). Selecton: A Server for Detecting Evolutionary Forces at a Single Amino-Acid Site. Bioinformatics.

[50] Kosakovsky Pond S.L., Posada D., Gravenor M.B., Woelk C.H., Frost S.D.W. (2006). Automated Phylogenetic Detection of Recombination Using a Genetic Algorithm. Mol. Biol. Evol..

[51] Sugiura N. (1978). Further Analysts of the Data by Akaike’s Information Criterion and the Finite Corrections: Further Analysts of the Data by Akaike’s. Commun. Stat. Methods.

[52] ur Rehman S., Nadeem A., Javed M., Hassan F., Luo X., Khalid R.B., Liu Q. (2020). Genomic Identification, Evolution and Sequence Analysis of the Heat-Shock Protein Gene Family in Buffalo. Genes.

[53] Wu S., Hassan F., Luo Y., Fatima I., Ahmed I., Ihsan A., Safdar W., Liu Q., ur Rehman S. (2021). Comparative Genomic Characterization of Buffalo Fibronectin Type III Domain Proteins: Exploring the Novel Role of FNDC5/Irisin as a Ligand of Gonadal Receptors. Biology.

[54] Rehman S.U., Hassan F., Luo X., Li Z., Liu Q. (2021). Whole-Genome Sequencing and Characterization of Buffalo Genetic Resources: Recent Advances and Future Challenges. Animals.

[55] Rehman S.U., Feng T., Wu S., Luo X., Lei A., Luobu B., Hassan F., Liu Q. (2021). Comparative Genomics, Evolutionary and Gene Regulatory Regions Analysis of Casein Gene Family in *Bubalus bubalis*. Front. Genet..

[56] Rehman S.U., Shafique L., Yousuf M.R., Liu Q., Ahmed J.Z., Riaz H. (2019). Spectrophotometric Calibration and Comparison of Different Semen Evaluation Methods in Nili-Ravi Buffalo Bulls. Pak. Vet. J.

[57] Hassan F., Nadeem A., Li Z., Javed M., Liu Q., Azhar J., Rehman M.S., Cui K., ur Rehman S. (2021). Role of Peroxisome Proliferator-Activated Receptors (PPARs) in Energy Homeostasis of Dairy Animals: Exploiting Their Modulation through Nutrigenomic Interventions. Int. J. Mol. Sci..

[58] Pontiggia A., Whitfield S., Goodfellow P.N., Lovell-Badge R., Bianchi M.E. (1995). Evolutionary Conservation in the DNA-Binding and -Bending Properties of HMG-Boxes from SRY Proteins of Primates. Gene.

[59] Yang J., Hu Y., Han J., Xiao K., Liu X., Tan C., Zeng Q., Du H. (2020). Genome-wide Analysis of the Chinese Sturgeon Sox Gene Family: Identification, Characterisation and Expression Profiles of Different Tissues. J. Fish Biol..

[60] Guruprasad K., Reddy B.V.B., Pandit M.W. (1990). Correlation between Stability of a Protein and Its Dipeptide Composition: A Novel Approach for Predicting in Vivo Stability of a Protein from Its Primary Sequence. Protein Eng. Des. Sel..

[61] Atsushi I. (1980). Thermostability and Aliphatic Index of Globular Proteins. J. Biochem..

[62] Mondal S.K., Sen M.K. (2019). An In-Silico Characterization of Sry-Related HMG Box C (SOXC) in Humans and Mouse. Meta Gene.

[63] Kyte J., Doolittle R.F. (1982). A Simple Method for Displaying the Hydropathic Character of a Protein. J. Mol. Biol..

[64] Magadum S., Banerjee U., Murugan P., Gangapur D., Ravikesavan R. (2013). Gene Duplication as a Major Force in Evolution. J. Genet..

[65] Lipinski K.J., Farslow J.C., Fitzpatrick K.A., Lynch M., Katju V., Bergthorsson U. (2011). High Spontaneous Rate of Gene Duplication in Caenorhabditis Elegans. Curr. Biol..

[66] Hurst L.D. (2002). The Ka/Ks Ratio: Diagnosing the Form of Sequence Evolution. Trends Genet. TIG.

[67] Wang Y., Feng L., Zhu Y., Li Y., Yan H., Xiang Y. (2015). Comparative Genomic Analysis of the WRKY III Gene Family in Populus, Grape, Arabidopsis and Rice. Biol. Direct.

[68] Consortium M.G.S., Waterston R.H., Lindblad-Toh K., Birney E., Rogers J., Abril J.F. (2002). Initial Sequencing and Comparative Analysis of the Mouse Genome. Nature.

[69] Kosakovsky Pond S.L., Posada D., Gravenor M.B., Woelk C.H., Frost S.D.W. (2006). GARD: A Genetic Algorithm for Recombination Detection. Bioinformatics.

[70] Yang Z. (2002). Inference of Selection from Multiple Species Alignments. Curr. Opin. Genet. Dev..

[71] Stange M., Sánchez-Villagra M.R., Salzburger W., Matschiner M. (2018). Bayesian Divergence-Time Estimation with Genome-Wide Single-Nucleotide Polymorphism Data of Sea Catfishes (Ariidae) Supports Miocene Closure of the Panamanian Isthmus. Syst. Biol..

[72] Carruthers T., Sun M., Baker W.J., Smith S.A., de Vos J.M., Eiserhardt W.L. (2022). The Implications of Incongruence between Gene Tree and Species Tree Topologies for Divergence Time Estimation. Syst. Biol..

[73] Wei L., Liu Y., Dubchak I., Shon J., Park J. (2002). Comparative Genomics Approaches to Study Organism Similarities and Differences. J. Biomed. Inform..

[74] Galperin M.Y., Koonin E.V. (2001). Comparative Genome Analysis. Methods Biochem. Anal..

[75] Sivashankari S., Shanmughavel P. (2007). Comparative Genomics-a Perspective. Bioinformation.

[76] Li W., Bickhart D.M., Ramunno L., Iamartino D., Williams J.L., Liu G.E. (2018). Genomic Structural Differences between Cattle and River Buffalo Identified through Comparative Genomic and Transcriptomic Analysis. Data Br..

[77] Dutta P., Talenti A., Young R., Jayaraman S., Callaby R., Jadhav S.K., Dhanikachalam V., Manikandan M., Biswa B.B., Low W.Y. (2020). Whole Genome Analysis of Water Buffalo and Global Cattle Breeds Highlights Convergent Signatures of Domestication. Nat. Commun..

[78] de Abreu Santos D.J., Ferreira de Camargo G.M., Cardoso D.F., Buzanskas M.E., Aspilcueta-Borquis R.R., Hurtado-Lugo N.A., de Araújo Neto F.R., Galvão de Albuquerque L., Ma L., Tonhati H. (2020). Linkage Disequilibrium-Based Inference of Genome Homology and Chromosomal Rearrangements between Species. G3 Genes Genomes Genet..

[79] Studer R.A., Dessailly B.H., Orengo C.A. (2013). Residue Mutations and Their Impact on Protein Structure and Function: Detecting Beneficial and Pathogenic Changes. Biochem. J..

[80] Loewe L. (2008). Genetic Mutation. Nat. Educ..

[81] Hartatik T., Bintara S., Ismaya I., Panjono P., Widyobroto B.P., Agus A., Budisatria I.G.S., Leroy P. (2020). Single Nucleotide Polymorphism of Sex Determining Region-Y Gene Coding Sequences in Belgian Blue Bull and Wagyu Bull Crossbred Cattle. Proceedings of the IOP Conference Series: Earth and Environmental Science.

[82] Berta P., Hawkins J.B., Sinclair A.H., Taylor A., Griffiths B.L., Goodfellow P.N., Fellous M. (1990). Genetic Evidence Equating SRY and the Testis-Determining Factor. Nature.

[83] Payen E.J., Cotinot C.Y. (1993). Comparative HMG-Box Sequences of the SRY Gene between Sheep, Cattle and Goats. Nucleic Acids Res..

[84] Cheng H., Shi H., Zhou R., Guo Y., Liu L., Liu J., Jiang Y., Kudo T., Sutou S. (2001). Characterization of Bovidae Sex-Determining Gene SRY. Genet. Sel. Evol..

[85] Xin C.A.I., ZHANG T.D.M. (2013). Unique Variations of SRY Gene Result in Distinct Patrilineal Phylogeny of Capra Hircus and Other Domestic Bovidae. Anim. Sci. Pap. Rep..

[86] Parma P., Feligini M., Greppi G., Enne G. (2004). The Complete Coding Region Sequence of River Buffalo (*Bubalus bubalis*) SRY Gene. DNA Seq..

[87] Sun T., Hanif Q., Chen H., Lei C., Dang R. (2019). Copy Number Variations of Four Y-Linked Genes in Swamp Buffaloes. Animals.

[88] Salehi L.B., Scarciolla O., Vanni G.F., Nardone A.M., Frajese G., Novelli G., Stuppia L. (2006). Identification of a Novel Mutation in the SRY Gene in a 46, XY Female Patient. Eur. J. Med. Genet..

[89] Brown S., Yu C., Lanzano P., Heller D., Thomas L., Warburton D., Kitajewski J., Stadtmauer L. (1998). A de Novo Mutation (Gln2Stop) at the 5’ End of the SRY Gene Leads to Sex Reversal with Partial Ovarian Function. Am. J. Hum. Genet..

[90] Zeng Y.T., Ren Z.R., Zhang M.L., Huang Y., Zeng F.Y., Huang S.Z. (1993). A New de Novo Mutation (A113T) in HMG Box of the SRY Gene Leads to XY Gonadal Dysgenesis. J. Med. Genet..

[91] Domenice S., Yumie Nishi M., Correia Billerbeck A.E., Latronico A.C., Aparecida Medeiros M., Russell A.J., Vass K., Marino Carvalho F., Costa Frade E.M., Prado Arnhold I.J. (1998). A Novel Missense Mutation (S18N) in the 5′ Non-HMG Box Region of the SRY Gene in a Patient with Partial Gonadal Dysgenesis and His Normal Male Relatives. Hum. Genet..

[92] Andonova S., Robeva R., Sirakov M., Mainhard K., Tomova A., Ledig S., Kumanov P., Savov A. (2015). A Novel SRY Gene Mutation p. F109L in a 46, XY Female with Complete Gonadal Dysgenesis. Sex. Dev..

[93] Wang X., Xue M., Zhao M., He F., Li C., Li X. (2018). Identification of a Novel Mutation (Ala66Thr) of SRY Gene Causes XY Pure Gonadal Dysgenesis by Affecting DNA Binding Activity and Nuclear Import. Gene.

[94] Cunha J.L., Soardi F.C., Bernardi R.D., Oliveira L.E.C., Benedetti C.E., Guerra-Junior G., Maciel-Guerra A.T., de Mello M.P. (2011). The Novel p. E89K Mutation in the SRY Gene Inhibits DNA Binding and Causes the 46, XY Disorder of Sex Development. Braz. J. Med. Biol. Res..

[95] Filges I., Kunz C., Miny P., Boesch N., Szinnai G., Wenzel F., Tschudin S., Zumsteg U., Heinimann K. (2011). A Novel Missense Mutation in the High Mobility Group Domain of SRY Drastically Reduces Its DNA-Binding Capacity and Causes Paternally Transmitted 46,XY Complete Gonadal Dysgenesis. Fertil. Steril..

[96] Helszer Z., Dmochowska A., Szemraj J., Słowikowska-Hilczer J., Wieczorek M., Jędrzejczyk S., Kałużewski B. (2013). A Novel Mutation (c. 341A>G) in the SRY Gene in a 46,XY Female Patient with Gonadal Dysgenesis. Gene.

[97] Hawkins J.R., Taylor A., Berta P., Levilliers J., Van der Auwera B., Goodfellow P.N. (1992). Mutational Analysis of SRY: Nonsense and Missense Mutations in XY Sex Reversal. Hum. Genet..

[98] Portnoi M.-F., Dumargne M.-C., Rojo S., Witchel S.F., Duncan A.J., Eozenou C., Bignon-Topalovic J., Yatsenko S.A., Rajkovic A., Reyes-Mugica M. (2018). Mutations Involving the SRY-Related Gene SOX8 Are Associated with a Spectrum of Human Reproductive Anomalies. Hum. Mol. Genet..

[99] Lamb A.N., Rosenfeld J.A., Neill N.J., Talkowski M.E., Blumenthal I., Girirajan S., Keelean-Fuller D., Fan Z., Pouncey J., Stevens C. (2012). Haploinsufficiency of SOX5 at 12p12. 1 Is Associated with Developmental Delays with Prominent Language Delay, Behavior Problems, and Mild Dysmorphic Features. Hum. Mutat..

[100] Li A., Hooli B., Mullin K., Tate R.E., Bubnys A., Kirchner R., Chapman B., Hofmann O., Hide W., Tanzi R.E. (2017). Silencing of the Drosophila Ortholog of SOX5 Leads to Abnormal Neuronal Development and Behavioral Impairment. Hum. Mol. Genet..

[101] Daoud H., Valdmanis P.N., Gros-Louis F., Belzil V., Spiegelman D., Henrion E., Diallo O., Desjarlais A., Gauthier J., Camu W. (2011). Resequencing of 29 Candidate Genes in Patients with Familial and Sporadic Amyotrophic Lateral Sclerosis. Arch. Neurol..

[102] Sun D., Yu Y., Zhang Y. (2006). A Y-Linked SNP in SRY Gene Differentiates Chinese Indigenous Swamp Buffalo and Introduced River Buffalo. Asian-Australas J. Anim. Sci..

