# Supplementary material for: Genome-Wide Identification, Evolutionary and Mutational Analysis of the Buffalo Sox Gene Family
Source: Animals (Basel). 2023 Jul 8;13(14):2246. doi: 10.3390/ani13142246 (PMC10376873; doi:10.3390/ani13142246)
Supplement: Supplementary file 1 [file animals-13-02246-s001.zip › animals-2335795-supplementary.pdf]

Supplementary Material

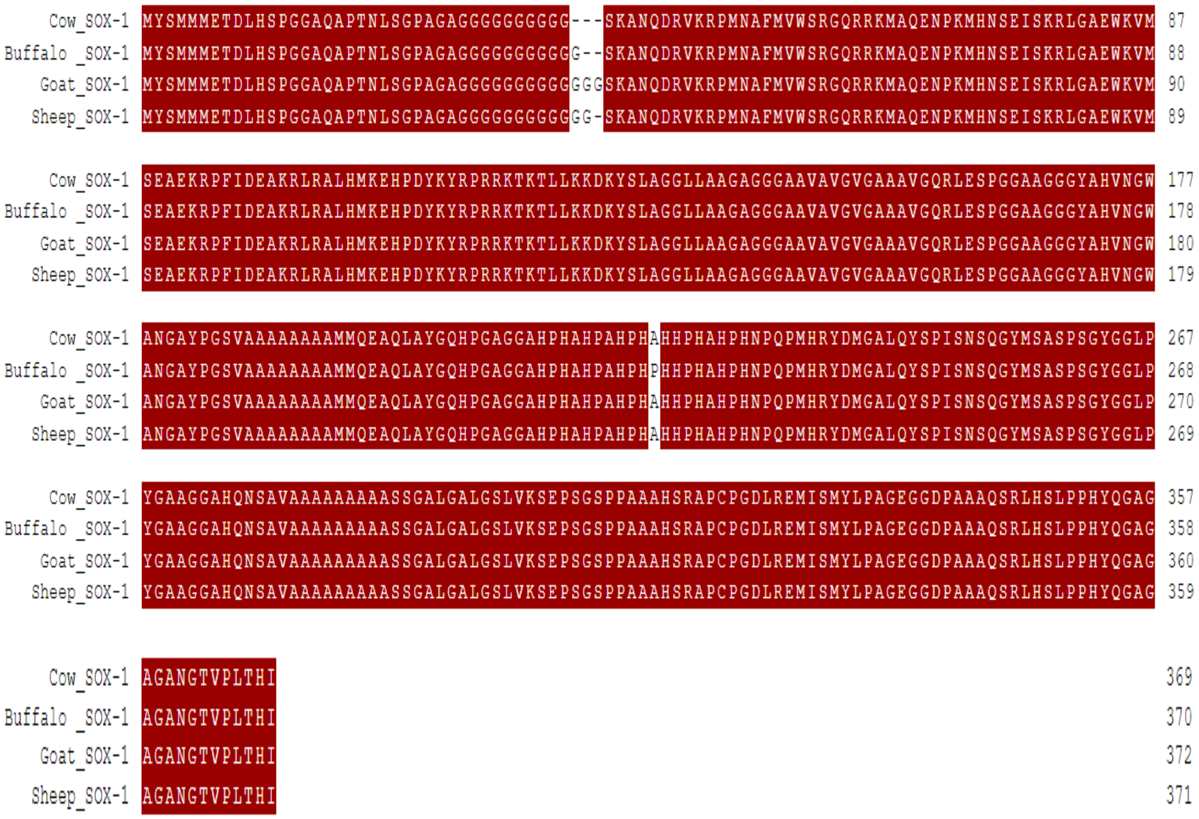

Figure S1. Comparative amino acid analysis of Sox1 gene in Cattle, buffalo, Sheep, and Goat

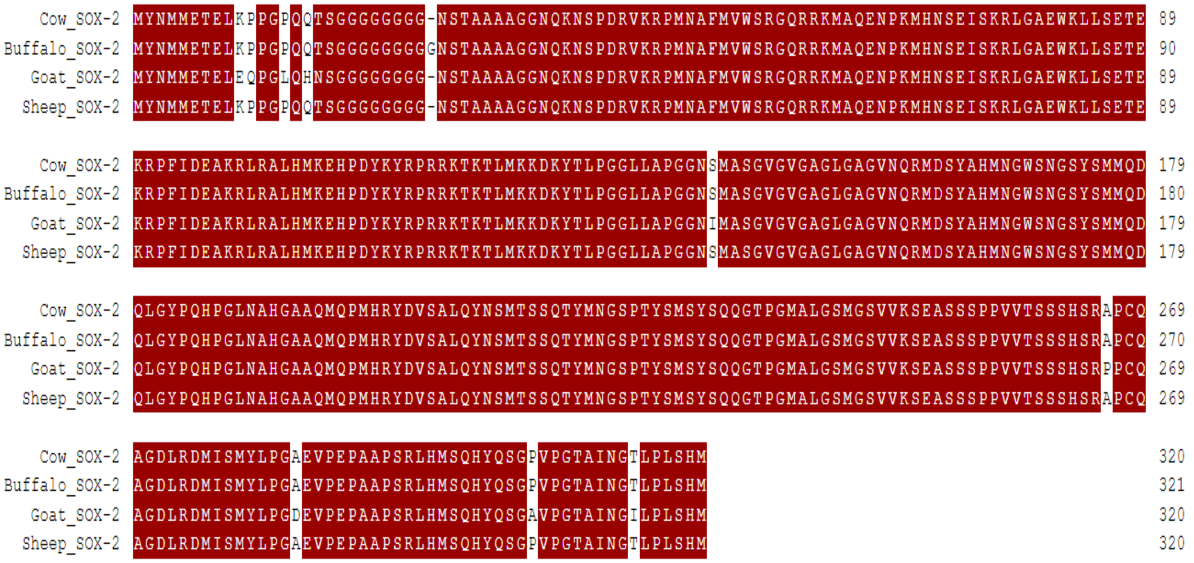

Figure S2. Comparative amino acid analysis of Sox2 gene in cattle, buffalo, sheep, and goat

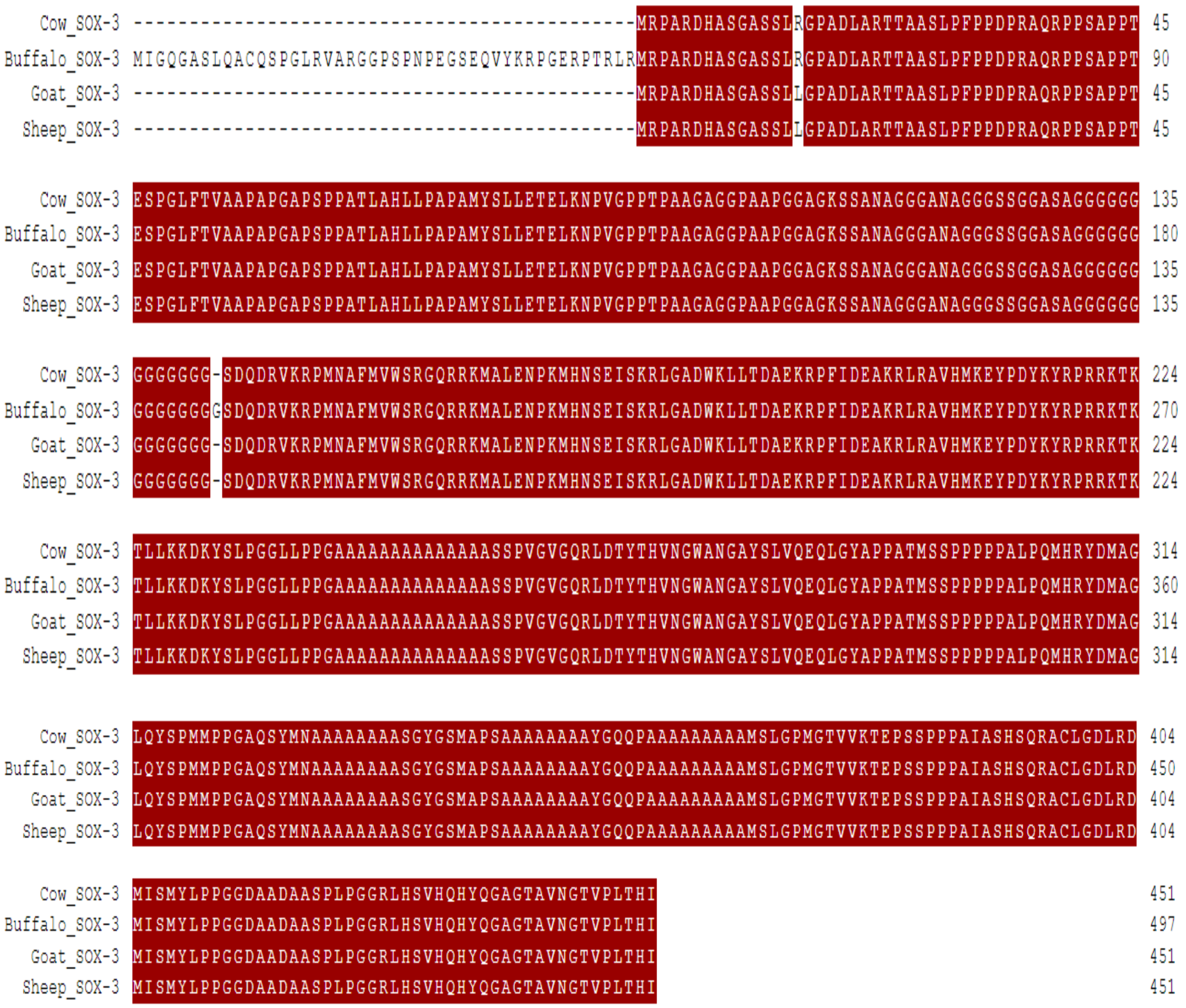

Figure S3. Comparative amino acid analysis of Sox3 gene in cattle, buffalo, sheep, and goat

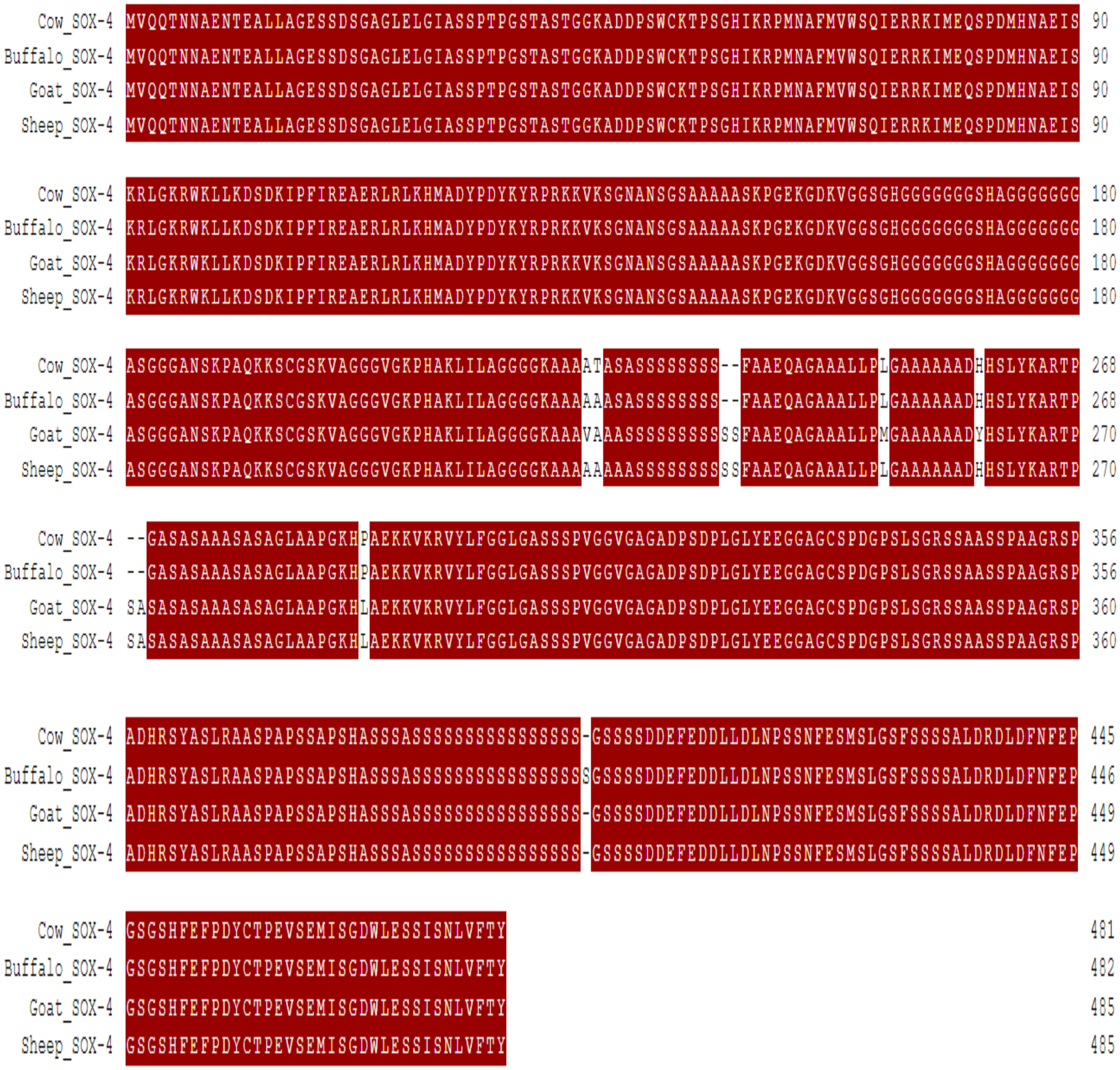

Figure S4. Comparative amino acid analysis of Sox4 gene in cattle, buffalo, sheep, and goat

```
Cow_SOX-5      MLTDPDLPQEFERMSSKRPASPYGEADGEVAMVTSRQKVEEEESDGLPAFHLPLHVSFPNKPHSEEFQPVSLLTQETCGHRTPASQHNTM  90
Buffalo_SOX-5  MLTDPDLPQEFERMSSKRPASPYGEADGEVAMVTSRQKVEEEESDGLPAFHLPLHVSFPNKPHSEEFQPVSLLTQETCGHRTPTSQHNTM  90
Goat_SOX-5     MLTDPDLPQEFERMSSKRPASPYGEADGEVAMVTSRQKVEEEESDGLPAFHLPLHVSFPNKPHSEEFQPVSLLTQETCGHRTPTSQHNTM  90
Sheep_SOX-5    MLTDPDLPQEFERMSSKRPASPYGEADGEVAMVTSRQKVEEEESDGLPAFHLPLHVSFPNKPHSEEFQPVSLLTQETCGHRTPTSQHNTM  90

Cow_SOX-5      EVDGNKVMSSFAPHNSSTSPQKAEEGGRQSGESLSSTTLGTPERRKGSLADVVDTLKQRKMEELIKNEPEETPSIEKLLSKDWKDKLLAM  180
Buffalo_SOX-5  EVDGNKVMSSFAPHNSSTSPQKAEEGGRQSGESLSSTTLGTPERRKGSLADVVDTLKQRKMEELIKNEPEETPSIEKLLSKDWKDKLLAM  180
Goat_SOX-5     EVDGNKVMSSFAPHNSSTSPQKAEEGGRQSGESLSSTTLGTPERRKGSLADVVDTLKQRKMEELIKNEPEETPSIEKLLSKDWKDKLLAM  180
Sheep_SOX-5    EVDGNKVMSSFAPHNSSTSPQKAEEGGRQSGESLSSTTLGTPERRKGSLADVVDTLKQRKMEELIKNEPEETPSIEKLLSKDWKDKLLAM  180

Cow_SOX-5      GSGNFGEIKGTPESLAEKERQLMGMINQLTSLREQLLAAHDEQKKLAASQIEKQRQQMELAKQQQEQIARQQQQLLQQQHKINLLQQQIQ  270
Buffalo_SOX-5  GSGNFGEIKGTPESLAEKERQLMGMINQLTSLREQLLAAHDEQKKLAASQIEKQRQQMELAKQQQEQIARQQQQLLQQQHKINLLQQQIQ  270
Goat_SOX-5     GSGNFGEIKGTPESLAEKERQLMGMINQLTSLREQLLAAHDEQKKLAASQIEKQRQQMELAKQQQEQIARQQQQLLQQQHKINLLQQQIQ  270
Sheep_SOX-5    GSGNFGEIKGTPESLAEKERQLMGMINQLTSLREQLLAAHDEQKKLAASQIEKQRQQMELAKQQQEQIARQQQQLLQQQHKINLLQQQIQ  270

Cow_SOX-5      QVQGQLPPLMIPVFPPDQRTLAAAAQQGFLLPPGFSYKAGCSDPYPVQLIPTTMAAAAAAATPGLGPLQLQQLYAAQLAAMQVSPGGKLPG  360
Buffalo_SOX-5  QVQGQLPPLMIPVFPPDQRTLAAAAQQGFLLPPGFSYKAGCSDPYPVQLIPTTMAAAAAAATPGLGPLQLQQLYAAQLAAMQVSPGGKLPG  360
Goat_SOX-5     -VQGQLPPLMIPVFPPDQRTLAAAAQQGFLLPPGFSYKAGCSDPYPVQLIPTTMAAAAAAATPGLGPLQLQQLYAAQLAAMQVSPGGKLPG  359
Sheep_SOX-5    QVQGQLPPLMIPVFPPDQRTLAAAAQQGFLLPPGFSYKAGCSDPYPVQLIPTTMAAAAAAATPGLGPLQLQQLYAAQLAAMQVSPGGKLPG  360

Cow_SOX-5      VPQGNLGAAVSPTSIHTDKSTNSPPPKSKDEVAQPLNLSAKPKTSDGKSPTSPTSPHMPALRINSGAGPLKASVPASLASPSARVSTIGY  450
Buffalo_SOX-5  VSQGNLGAAVSPTSIHTDKSTNSPPPKSKDEVAQPLNLSAKPKTSDGKSPTSPTSPHMPALRINSGAGPLKASVPASLASPSARVSTIGY  450
Goat_SOX-5     VSQGNLGAAVSPTSIHTDKSTNSPPPKSKDEVAQPLNLSAKPKTSDGKSPTSPTSPHMPALRINSGAGPLKASVPASLASPSARVSTIGY  449
Sheep_SOX-5    VSQGNLGAAVSPTSIHTDKSTNSPPPKSKDEVAQPLNLSAKPKTSDGKSPTSPTSPHMPALRINSGAGPLKASVPASLASPSARVSTIGY  450

Cow_SOX-5      LNDHDAVTKAIQEARQMKEQLRREQQVLDGKVAVVNSLGLNNCRTEKEKTTLESLTQQLAVKQNEEGKFSHAMMDFNMSGDSDGSAGVSE  540
Buffalo_SOX-5  LNDHDAVTKAIQEARQMKEQLRREQQVLDGKVAVVNSLGLNNCRTEKEKTTLESLTQQLAVKQNEEGKFSHAMMDFNMSGDSDGSAGVSE  540
Goat_SOX-5     LNDHDAVTKAIQEARQMKEQLRREQQVLDGKVAVVNSLGLNNCRTEKEKTTLESLTQQLAVKQNEEGKFSHAMMDFNMSGDSDGSAGVSE  539
Sheep_SOX-5    LNDHDAVTKAIQEARQMKEQLRREQQVLDGKVAVVNSLGLNNCRTEKEKTTLESLTQQLAVKQNEEGKFSHAMMDFNMSGDSDGSAGVSE  540

Cow_SOX-5      SRIYRESRGRGSNEPHIKRPMNAFMVWAKDERRKILQAFPDMHNSNISKILGSRWKAMTNLEKQPYYEEQARLSKQHLEKYPDYKYKPRP  630
Buffalo_SOX-5  SRIYRESRGRGSNEPHIKRPMNAFMVWAKDERRKILQAFPDMHNSNISKILGSRWKAMTNLEKQPYYEEQARLSKQHLEKYPDYKYKPRP  630
Goat_SOX-5     SRIYRESRGRGSNEPHIKRPMNAFMVWAKDERRKILQAFPDMHNSNISKILGSRWKAMTNLEKQPYYEEQARLSKQHLEKYPDYKYKPRP  629
Sheep_SOX-5    SRIYRESRGRGSNEPHIKRPMNAFMVWAKDERRKILQAFPDMHNSNISKILGSRWKAMTNLEKQPYYEEQARLSKQHLEKYPDYKYKPRP  630

Cow_SOX-5      KRTCLVDGKKLRIGEYKAIMRNRRQEMRQYFNVGQQAQIPIATAGVVYPGAIAMAGMPSPHLPSEHSSVSSSPEPGMPVIQSTYGVKGEE  720
Buffalo_SOX-5  KRTCLVDGKKLRIGEYKAIMRNRRQEMRQYFNVGQQAQIPIATAGVVYPGAIAMAGMPSPHLPSEHSSVSSSPEPGMPVIQSTYGVKGEE  720
Goat_SOX-5     KRTCLVDGKKLRIGEYKAIMRNRRQEMRQYFNVGQQAQIPIATAGVVYPGAIAMAGMPSPHLPSEHSSVSSSPEPGMPVIQSTYGVKGEE  719
Sheep_SOX-5    KRTCLVDGKKLRIGEYKAIMRNRRQEMRQYFNVGQQAQIPIATAGVVYPGAIAMAGMPSPHLPSEHSSVSSSPEPGMPVIQSTYGVKGEE  720

Cow_SOX-5      PHIKEEIQAEDINGEIYDEYDEEEDDPDVDYGSDSENHIAGQAN  764
Buffalo_SOX-5  PHIKEEIQAEDINGEIYDEYDEEEDDPDVDYGSDSENHIAGQAN  764
Goat_SOX-5     PHIKEEIQAEDINGEIYDEYDEEEDDPDVDYGSDSENHIAGQAN  763
Sheep_SOX-5    PHIKEEIQAEDINGEIYDEYDEEEDDPDVDYGSDSENHIAGQAN  764
```

Figure S5. Comparative amino acid analysis of Sox5 gene in cattle, buffalo, sheep, and goat

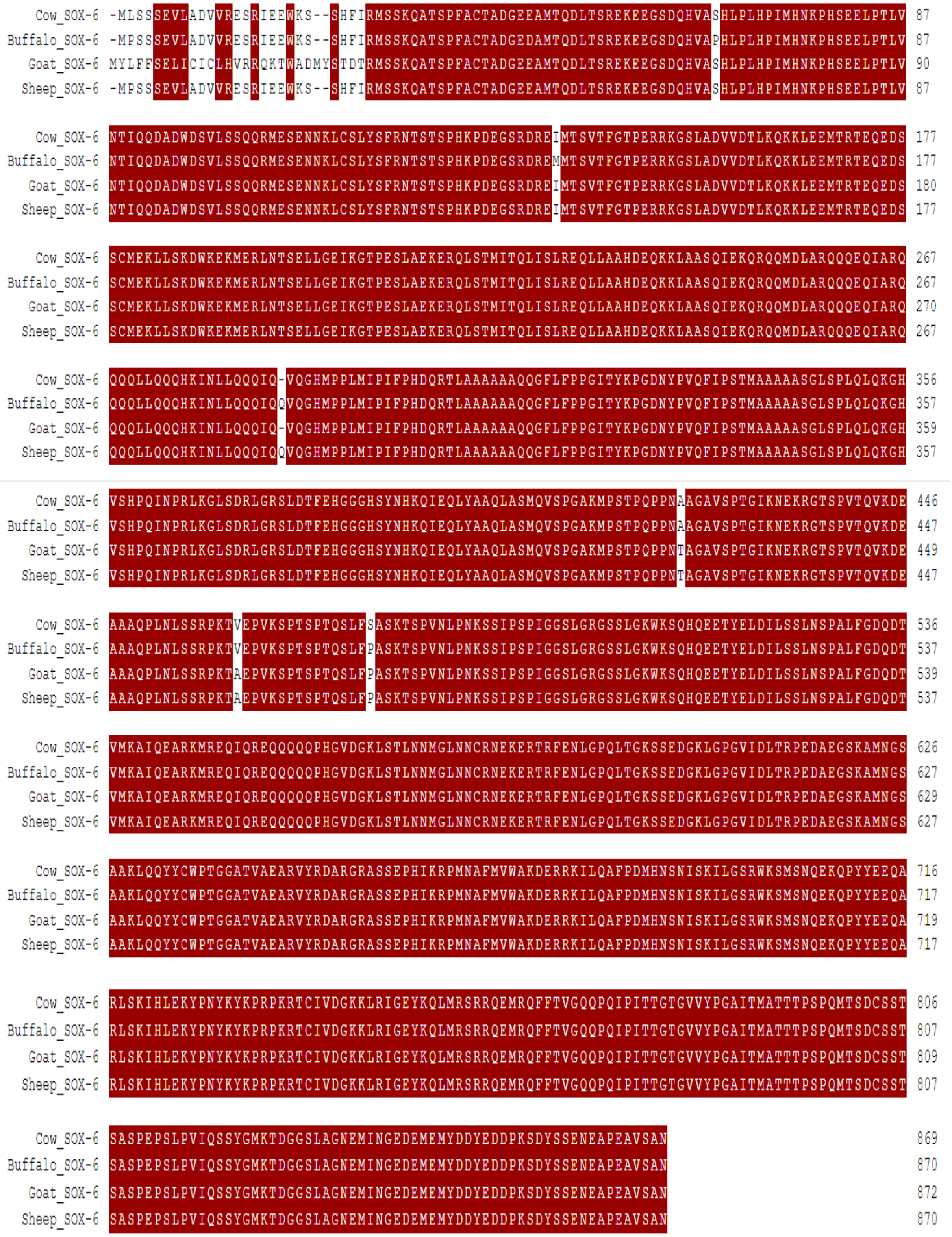

Figure S6. Comparative amino acid analysis of Sox6 gene in cattle, buffalo, sheep, and goat

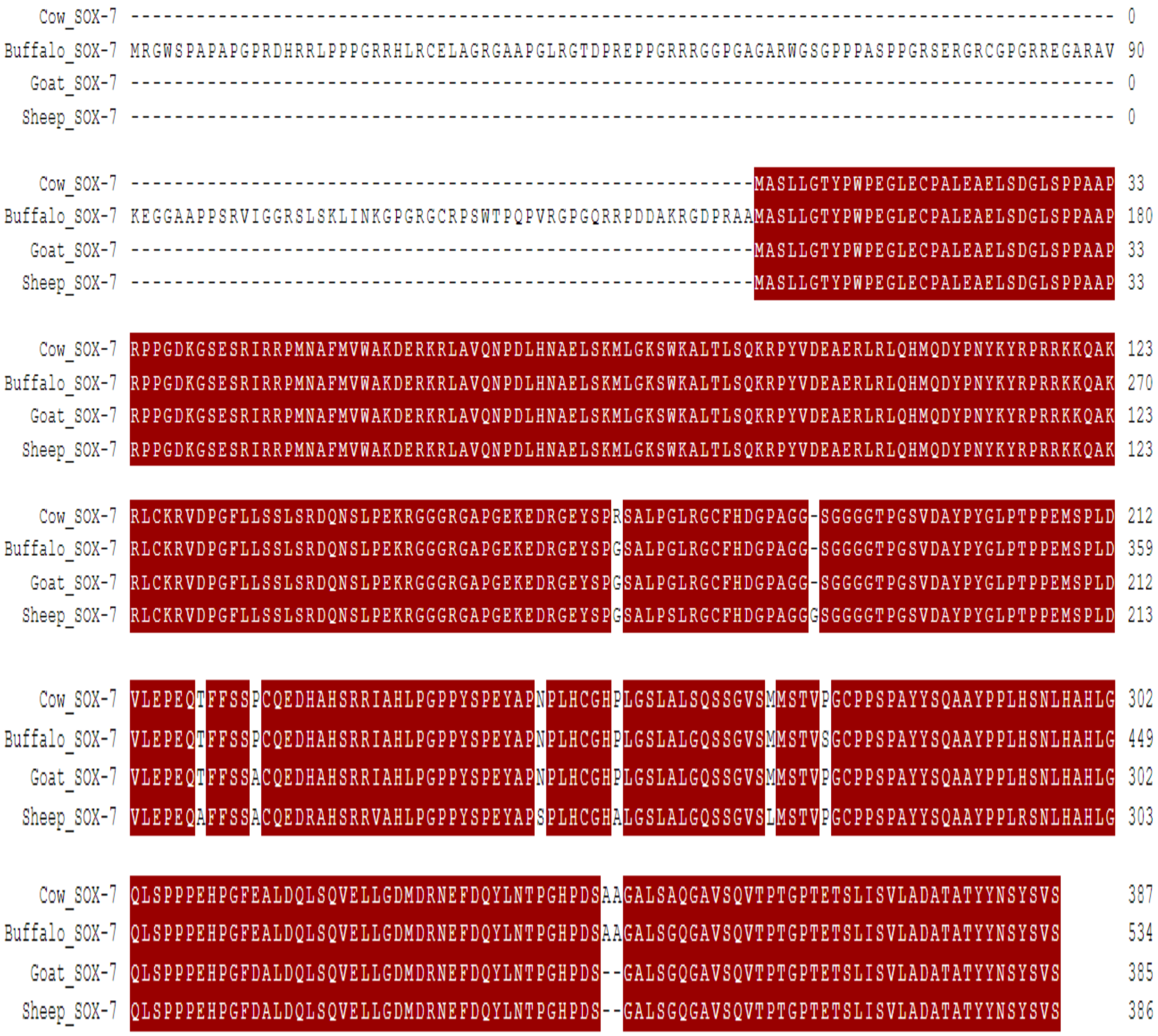

Figure S7. Comparative amino acid analysis of Sox7 gene in cattle, buffalo, sheep, and goat

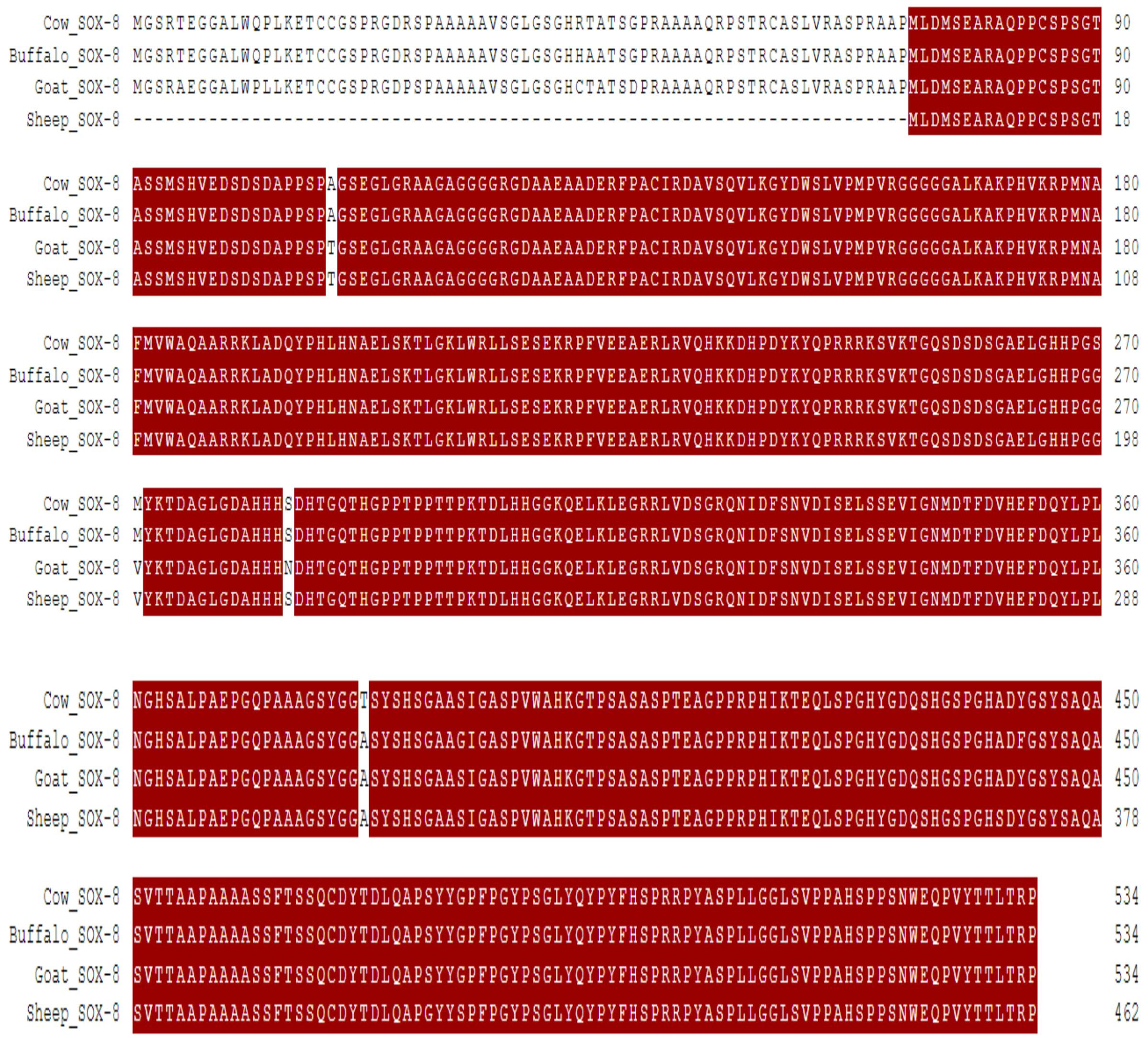

Figure S8. Comparative amino acid analysis of Sox8 gene in cattle, buffalo, sheep, and goat

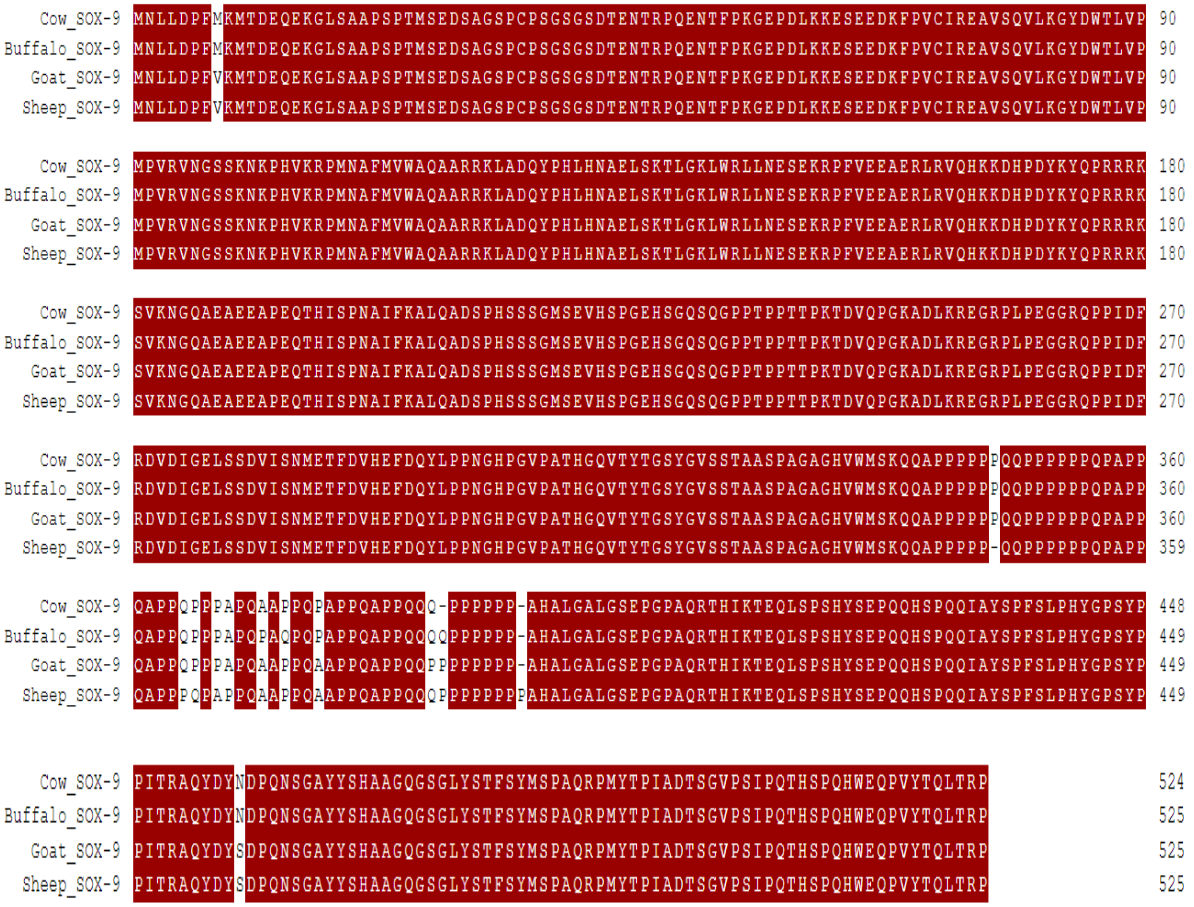

Figure S9. Comparative amino acid analysis of Sox9 gene in cattle, buffalo, sheep, and goat

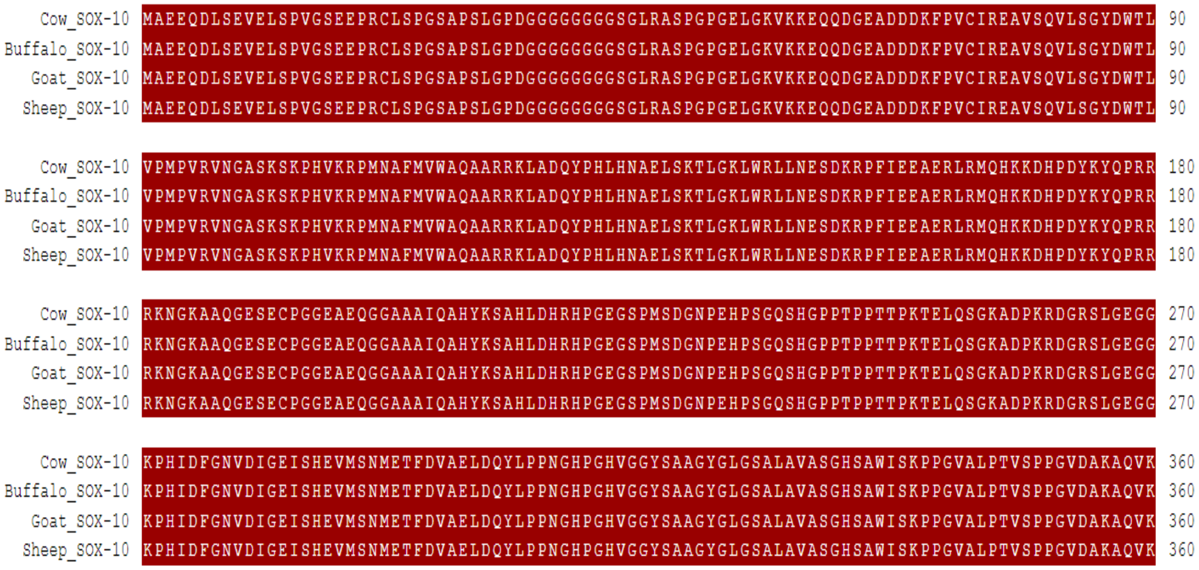

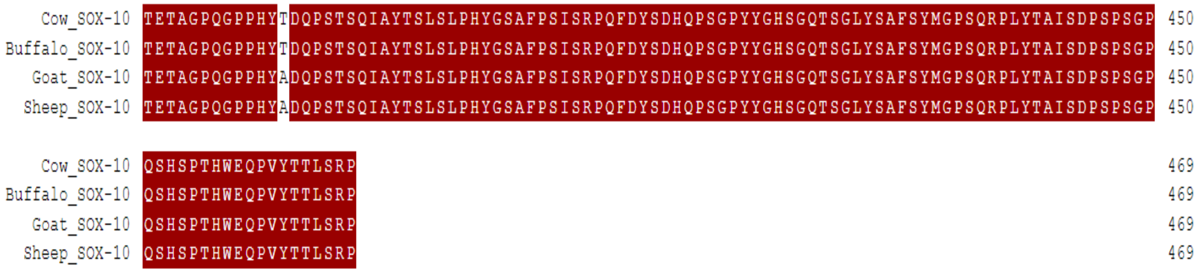

Figure S10. Comparative amino acid analysis of Sox10 gene in cattle, buffalo, sheep, and goat

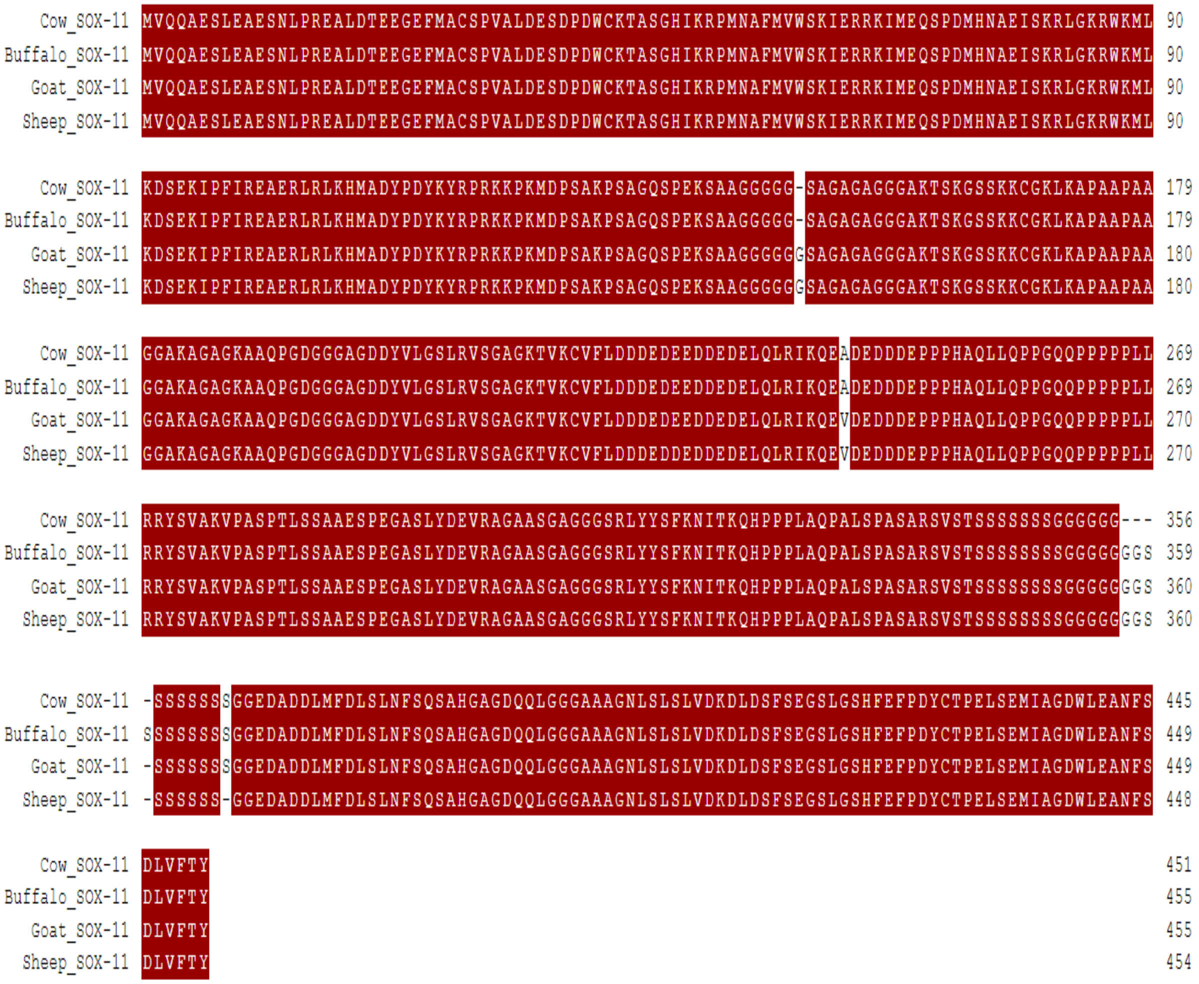

Figure S11. Comparative amino acid analysis of Sox11 gene in cattle, buffalo, sheep, and goat

```
     Cow_SOX-12 MVQQRGARAKRDGGPPPPGPGPAEEGAREPGWCKTPSGHIKRPMNAFMVWSQHERRKIMDQWPDMHNAEISKRLGRRWQLLQDSEKIPFV 90
 Buffalo_SOX-12 MVQQRGARAKRDGGPPPPGPGPAEEGAREPGWCKTPSGHIKRPMNAFMVWSQHERRKIMDQWPDMHNAEISKRLGRRWQLLQDSEKIPFV 90
    Goat_SOX-12 MVQQRGARAKRDGGPPPPGPGPAEEGAREPGWCKTPSGHIKRPMNAFMVWSQHERRKIMDQWPDMHNAEISKRLGRRWQLLQDSEKIPFV 90
   Sheep_SOX-12 MVQQRGARAKRDGGPPPPGPGPAEEGAREPGWCKTPSGHIKRPMNAFMVWSQHERRKIMDQWPDMHNAEISKRLGRRWQLLQDSEKIPFV 90

     Cow_SOX-12 REAERLRLKHMADYPDYKYRPRKKSKGAPAKARPRPPGGGGGGSRLKPGPQLPGRGGRRAAGGPLGGGAAAPEDDDDEDDEELLEVRLVE 180
 Buffalo_SOX-12 REAERLRLKHMADYPDYKYRPRKKSKGAPAKARPRPPGGGGGGSRLKPGPQLPGRGGRRAAGGPLGGGAAAPEDDDDEDDEELLEVRLVE 180
    Goat_SOX-12 REAERLRLKHMADYPDYKYRPRKKSKGAPAKARPRPPGGGGGGSRLKPGPQLPGRGGRRAAGGPLGGGAAAPEDDDDEDDEELLEVRLVE 180
   Sheep_SOX-12 REAERLRLKHMADYPDYKYRPRKKSKGAPAKARPRPPGGGGGGSRLKPGPQLPGRGGRRAAGGPLGGGAAAPEDDDDEDDEELLEVRLVE 180

     Cow_SOX-12 TPGRELWRMVPAGRAARGPAERAQGPSGEGAAVTAASPTPSEDEEPEEEEEEAAAAEEGEEEKVASGEEPLGFLSRGPPGPAGLDCSALD 270
 Buffalo_SOX-12 TPGRELWRMVPAGRAARGPAERAQGPSGEGAAVTAASPTPSEDEEPEEEEEEAAAAEEGEEEKVASGEEPLGFLSRGPPGPAGLDCSALD 270
    Goat_SOX-12 TPGRELWRMVPAGRAARGPAERAQGPSGEGAAVTAASPTPSEDEEPEEEEEEAAAAEEGEEEKVASGEEPLGFLSRGPPGPAGLDCSALD 270
   Sheep_SOX-12 TPGRELWRMVPAGRAARGPVERAQGPSGEGAAVTAASPTPSEDEEPEEEEEEAAAAEEGEEEKVASGEEPLGFLSRGPPGPAGLDCSALD 270

     Cow_SOX-12 RDPDLPPPSGTSHFEFPDYCTPEVTEMIAGDWRPSSIADLVFTY 314
 Buffalo_SOX-12 RDPDLPPPSGTSHFEFPDYCTPEVTEMIAGDWRPSSIADLVFTY 314
    Goat_SOX-12 RDPDLPPPSGTSHFEFPDYCTPEVTEMIAGDWRPSSIADLVFTY 314
   Sheep_SOX-12 RDPDLPPPSGTSHFEFPDYCTPEVTEMIAGDWRPSSIADLVFTY 314
```

Figure S12. Comparative amino acid analysis of Sox12 gene in cattle, buffalo, sheep, and goat

```
     Cow_SOX-13 MERPGAAPAAPPPPGWPPRFPGFLTSPRSWKQAPFLHPFLMMSMRSPGSAQLVLDEVGTMVNCTVKAEGKKEPCYEDPQGSATAAESQPG 90
 Buffalo_SOX-13 MERPRAAAAAPPPPGWPPRVPGFLTSPRSWKQAPFLHPLLMMSMRSPGSAQLVLDEVGTMVNCTVKAEGKKEPCYEDPQGSAAAAESQPG 90
    Goat_SOX-13 -----------------------------------------MSMRSPGSAQLVLDEVGTMVNCSVKAEGKKEPCYEDPQGSATAAELQPG 49
   Sheep_SOX-13 MERAGAAPAAPPPPGWPPRFPGFLTSPRSWKQAPFLHPLLMMSMRSPGSAQLVLDEVGTMVNCSVKAEGKKEPCYEDPQGSATAAESQPG 90

     Cow_SOX-13 DPARTPQNGADPQAPTQGDRDIQCVSQDSSSLENSESPEPRRPGGSEAASGSQEKLDFNRNLKEVVPAIEKLLSSGWKEKFLGRSSVETK 180
 Buffalo_SOX-13 DPARIPQNGADPQAPAQGDRDIQCVLQDSSSLENSESPEPRRPGGSEAASGSQEKLDFNRNLKEVVPAIEKLLSSDWKEKFLGRSSVETK 180
    Goat_SOX-13 DPARPAQNGADLQAPAQ----------DSSSLENSGSPEPRRSGGSEAASGSQEKLDFNRNLKEVVPAIEKLLSSDWKEKFLGRSSVETK 129
   Sheep_SOX-13 DPARPAQNGADLQAPAQ----------DSSSPENSGSPEPRRPGGSEAASGSQEKLDFNRNLKEVVPAIEKLLSSDWKEKFLGRSSVETK 170

     Cow_SOX-13 DVKGTKESLAEKELQLLVMIHQLSALRDQLLTAHSEQKNMAAMLLEKQQQQMELARQQQEQIAKQQQQLIQQQHKINLLQQQIQQVNMPY 270
 Buffalo_SOX-13 DVKGTKESLAEKELQLLVMIHQLSALRDQLLTAHSEQKNMAAMLLEKQQQQMELARQQQEQIAKQQQQLIQQQHKINLLQQQIQQVNMPY 270
    Goat_SOX-13 DVKGTKESLAEKELQLLVMIHQLSALRDQLLTAHSEQKNMAAMLLEKQQQQMELARQQQEQIAKQQQQLIQQQHKINLLQQQIQQVNMPY 219
   Sheep_SOX-13 DVKGTKESLAEKELQLLVMIHQLSALRDQLLTAHSEQKNMAAMLLEKQQQQMELARQQQEQIAKQQQQLIQQQHKINLLQQQIQQVNMPY 260

     Cow_SOX-13 VMIPAFPPSHQPLPVTPDSQLALPIQPIPCKPVEYPLQLLHSPPAPVVKRPGALPAHHPLQEPSQPLNLTAKPKASELPNSSSSPNLKLS 360
 Buffalo_SOX-13 VMIPAFPPSHQPLPVTPDSQLALPIQPIPCKPVEYPLQLLHSPPTPVVKRPGALPAHHPLQEPSQPLNLTAKPKASELPNSSSSPNLKLS 360
    Goat_SOX-13 VMIPAFPPSHQPLPVTPDSQLALPIQPIPCKPVEYPLQLLHSPPAPVVKRPGPLSAHHPLQEPSQPLNLTAKPKASELPNSSSSPNLKLS 309
   Sheep_SOX-13 VMIPAFPPGHQPLPVTPDSQLALPIQPIPCKPVEYPLQLLHSPPAPVVKRPGPLSAHHPLQEPSQPLNLTAKPKASELPNSSSSPNLKLS 350
```

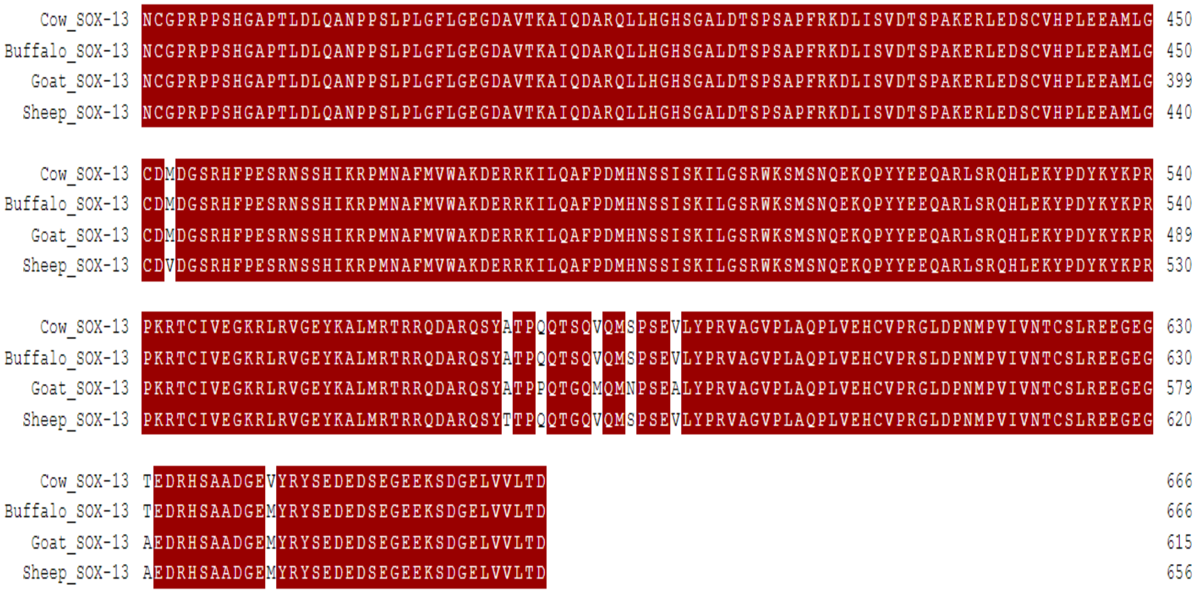

Figure S13. Comparative amino acid analysis of Sox13 gene in cattle, buffalo, sheep, and goat

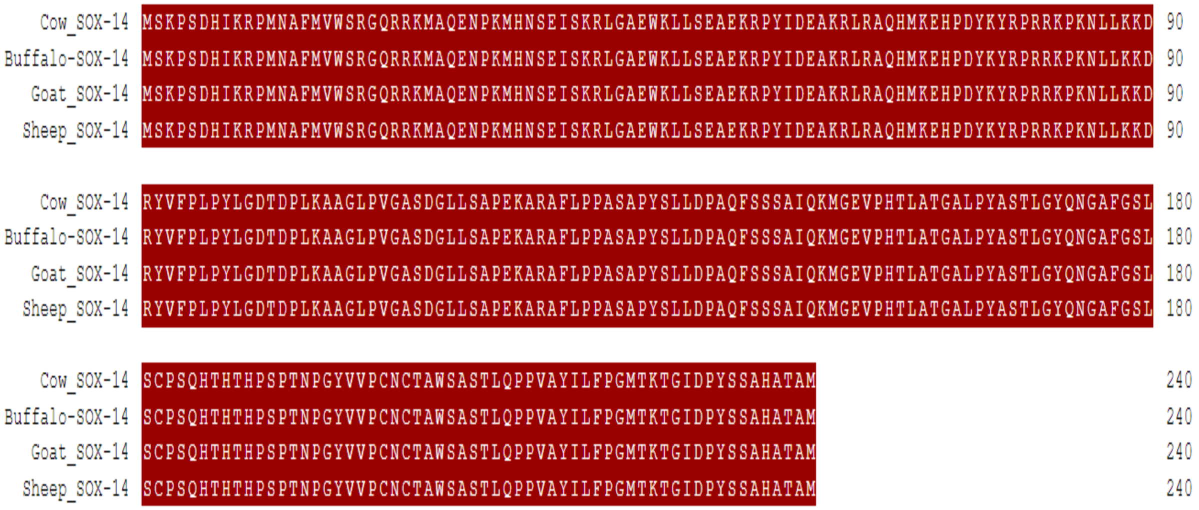

Figure S14. Comparative amino acid analysis of Sox14 gene in cattle, buffalo, sheep, and goat

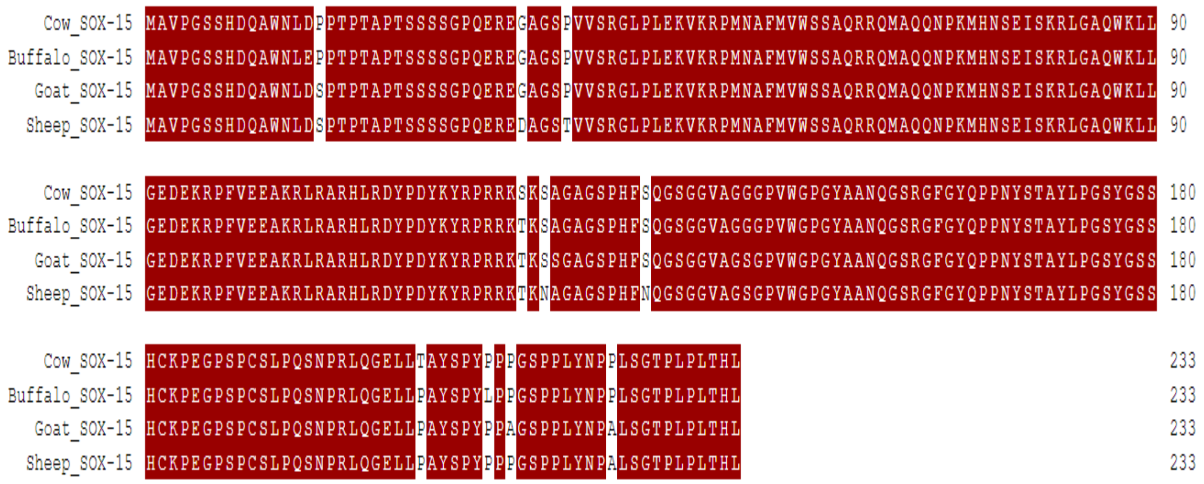

Figure S15. Comparative amino acid analysis of Sox15 gene in cattle, buffalo, sheep, and goat

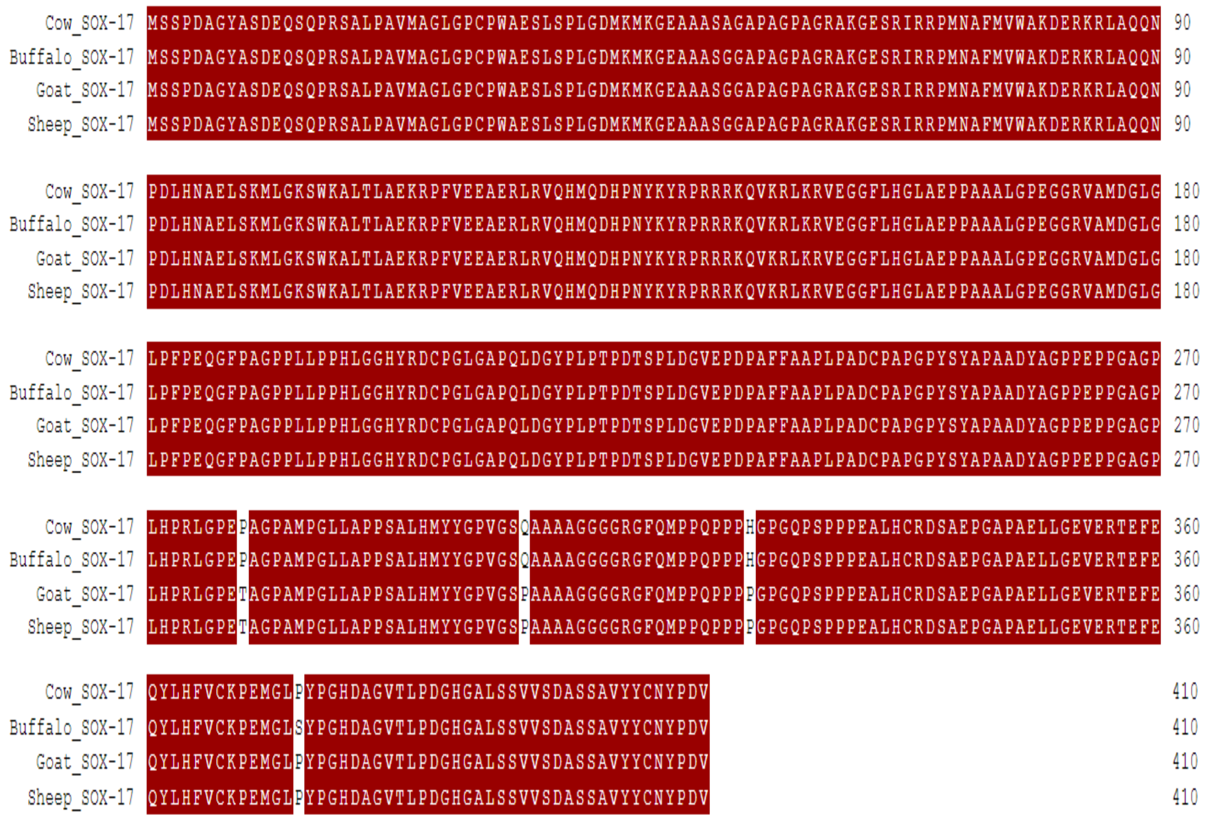

Figure S16. Comparative amino acid analysis of Sox17 gene in cattle, buffalo, sheep, and goat

```
     Cow_SOX-18 MQRSPLGYGAQDDPPARRDCAWAPGPGAAAEPRGLPAVPVVP---TAPAAPASPPSPPRSPPRSPEPGRYGLSPAGRGERQGTDESRIRR 87
 Buffalo_SOX-18 MQRSPLGYGAQDDPPARRDCAWAPGPGAAAEPRGLPAVPVVP---TAPAAPASPPSPPRSPPRSPEPGRYGLSPAGRGERQGTDESRIRR 87
    Goat_SOX-18 MQRSPLGYGAQDDPPARRDCAWAPGPGAAAEPRGLPAVPAVPAVPTAPAAPASPPSPPRSPPRSPEPGRYGLSPAGRGERQGTDESRIRR 90
   Sheep_SOX-18 MQRSPLGYGAQDDPPARRDCAWAPGPGAAAEPRGLPAVPAVP---TAPAAPASPPSPPRSPPRSPEPGRYGLSPAGRGERQGTDESRIRR 87

     Cow_SOX-18 PMNAFMVWAKDERKRLAQQNPDLHNAVLSKMLGKAWKELSPAEKRPFVEEAERLRVQHLRDHPNYKYRPRRKKQARKARRLEPGLLLPGL 177
 Buffalo_SOX-18 PMNAFMVWAKDERKRLAQQNPDLHNAVLSKMLGKAWKELSPAEKRPFVEEAERLRVQHLRDHPNYKYRPRRKKQARKARRLEPGLLLPGL 177
    Goat_SOX-18 PMNAFMVWAKDERKRLAQQNPDLHNAVLSKMLGKAWKELSPAEKRPFVEEAERLRVQHLRDHPNYKYRPRRKKQARKARRLESGLLLPGL 180
   Sheep_SOX-18 PMNAFMVWAKDERKRLAQQNPDLHNAVLSKMLGKAWKELSPAEKRPFVEEAERLRVQHLRDHPNYKYRPRRKKQARKARRLESGLLLPGL 177

     Cow_SOX-18 APPPPPPPPE--PFPAATGPARVFRELPPLGAEFDGLGLPTPERSPLDGLEPGEAAFFPPPAAPEDCSLRAFRAPYGPAELPRNPGGCFG 265
 Buffalo_SOX-18 APPPPPPPPE--PFPAATGPARVFRELPPLGAEFDGLGLPTPERSPLDGLEPGEAAFFPPPAAPEDCSLRAFRAPYGPAELPRNPGGCFG 265
    Goat_SOX-18 AAPPPPPPPP-EPFPAATGPARVFRELPPLGAEFDGLGLPTPERSPLDGLEPGEAAFFPPPAAPEDCSLRAFRAPYGPAELPRNPGGCFG 269
   Sheep_SOX-18 AAPPPPPPPPPEPFPAATGPARVFRELPPLGAEFDGLGLPTPERSPLDGLEPGEAAFFPPPAAPEDCSLRAFRAPYGPAELPRNPGGCFG 267

     Cow_SOX-18 APPAEALRTAPGPAAPLCGLYYSAPGAPGPGP--YPGPLSPPPEAPPLESAEPLGPAADLWADVDLTEFDQYLNCSRTRPDAAGLPYHVA 353
 Buffalo_SOX-18 APPAEALRTAPGPAAPLCGLYYSAPGAPGPGPGPYPGPLSPPPEAPPLESVEPLGPAADLWADVDLTEFDQYLNCSRTRPDAAGLPYHVA 355
    Goat_SOX-18 APPAEALRTAPGPAAQLCGLYYSAPGAPGPGP--YPGPLSPPPEAPPLESAEPLGPAADLWADVDLTEFDQYLNCSRTRPDAAGLPYHVA 357
   Sheep_SOX-18 APPAEALRTAPGPAAQLCGLYYSAPGAPGPGP--YPGPLSPPPEAPPLESAEPLGPAADLWADVDLTEFDQYLNCSRTRPDAAGLPYHVA 355

     Cow_SOX-18 LAKLAPRTMSCPEESSLIAALSDASSAVYYSACISG 389
 Buffalo_SOX-18 LAKLAPRTMTCPEESSLIAALSDASSAVYYSACISG 391
    Goat_SOX-18 LAKLAPRTMSCPEESSLIAALSDASSAVYYSACISG 393
   Sheep_SOX-18 LAKLAPRTMSCPEESSLIAALSDASSAVYYSACISG 391
```

Figure S17. Comparative amino acid analysis of Sox18 gene in cattle, buffalo, sheep, and goat

```
     Cow_SOX-21 MSKPVDHVKRPMNAFMVWSRAQRRKMAQENPKMHNSEISKRLGAEWKLLTESEKRPFIDEAKRLRAMHMKEHPDYKYRPRRKPKTLLKKD 90
 Buffalo_SOX-21 MSKPVDHVKRPMNAFMVWSRAQRRKMAQENPKMHNSEISKRLGAEWKLLTESEKRPFIDEAKRLRAMHMKEHPDYKYRPRRKPKTLLKKD 90
    Goat_SOX-21 MSKPVDHVKRPMNAFMVWSRAQRRKMAQENPKMHNSEISKRLGAEWKLLTESEKRPFIDEAKRLRAMHMKEHPDYKYRPRRKPKTLLKKD 90
   Sheep_SOX-21 MSKPVDHVKRPMNAFMVWSRAQRRKMAQENPKMHNSEISKRLGAEWKLLTESEKRPFIDEAKRLRAMHMKEHPDYKYRPRRKPKTLLKKD 90

     Cow_SOX-21 KFAFPVPYGLGGVADAEHPALKAGAGLHAGAGSGGLVPESLLANPEKAAAAAAAAAARVFFPQSAAAAAAAAAAAAAGSPYSLLDLGSKM 180
 Buffalo_SOX-21 KFAFPVPYGLGGVADAEHPALKAGAGLHTGAGSGGLVPESLLANPEKAAAAAAAAAARVFFPQSAAAAAAAAAAAAAGSPYSLLDLGSKM 180
    Goat_SOX-21 KFAFPVPYGLGGVADAEHPALKAGAGLHAGAGSGGLVPESLLANPEKAAAAAAAAAARVFFPQSAAAAAAAAAAAAAGSPYSLLDLGSKM 180
   Sheep_SOX-21 KFAFPVPYGLGGVADAEHPALKAGAGLHAGAGSGGLVPESLLANPEKAAAAAAAAAARVFFPQSAAAAAAAAAAAAAGSPYSLLDLGSKM 180

     Cow_SOX-21 AEISSSSSGLPYASSLGYPTAGAGAFHGAAAAAAAAAAAAGGHTHSHPSPGNPGYMIPCNCSAWPSPGLQPPLAYILLPGMGKPQLDPYP 270
 Buffalo_SOX-21 AEISSSSSGLPYASSLGYPTAGAGAFHGAAAAAAAAAAAAGGHTHSHPSPGNPGYMIPCNCSAWPSPGLQPPLAYILLPGMGKPQLDPYP 270
    Goat_SOX-21 AEISSSSSGLPYASSLGYPTAGAGAFHGAAAAAAAAAAAAGGHTHSHPSPGNPGYMIPCNCSAWPSPGLQPPLAYILLPGMGKPQLDPYP 270
   Sheep_SOX-21 AEISSSSSGLPYASSLGYPTAGAGAFHGAAAAAAAAAAAAGGHTHSHPSPGNPGYMIPCNCSAWPSPGLQPPLAYILLPGMGKPQLDPYP 270

     Cow_SOX-21 AAYAAAL 277
 Buffalo_SOX-21 AAYAAAL 277
    Goat_SOX-21 AAYAAAL 277
   Sheep_SOX-21 AAYAAAL 277
```

Figure S18. Comparative amino acid analysis of Sox21 gene in cattle, buffalo, sheep, and goat

```
Cow_SOX-30      MERARPEQPPQQRQLPRATPPRPLRPAPPPLPVEGASFRAAATEPSPSPPTPCAAAIATVASSCGEPSASGVQPAARRLLQVKPEQVLLL  90
Buffalo_SOX-30  MERARPEQPPQQRQLPRATPSRPLRPAAPPLPVEGASFRAAVTEPSPSPPTPWAAAIATVASSCGEPSASGVQPAARRLLQVKPEQVLLL  90
Goat_SOX-30     MERARPEQPLQQRQLPRATPPRPLRPAPPSLPVEGASFRAAAAEPSPSPPTPCAAAIATVASSCGEPSALSVQPAARRLLQVKPEQVLLL  90
Sheep_SOX-30    MERARPEQPLQQRQLPRATPPRPLRPAPPSLPVEGASFRAAAAEPSPSPPTPCAAAIATVASSCGEPSALSVQPAARRLLQVKPEQVLLL  90

Cow_SOX-30      PPGPPLPQAREESAATSPAQARLLQLRPELLLLPPPPPASEGIPCRPELHPLQPRALHVKAEKQEPGPGLDLLAGPRRAVEACPKTSRTV  180
Buffalo_SOX-30  PPGPPLPQAREEGAATSPAQARLLQLRSELLLLPPPPPASEGIPCRPELHPLQPRALHVKAEKQEPGPGLDLLAGPRRAVEACPKTSRRV  180
Goat_SOX-30     PPGPPLPQAREEGAATSPAQARLLQLRPELLLLPPPPPASEGVPCRPELHPLQPRALHVKAEKQEPGPGLDLLAGPRRAVEACSKTSRTV  180
Sheep_SOX-30    PPGPPLPQAREEGAATSPAQARLLQLRPELLLLPPPPPASEGVPCRPELHPLQPRALHVKAEKQDPGPGLDLLAGPRRAVEACPKTSRTV  180

Cow_SOX-30      KAEGSGPLNSRRGEEKKGKLEAEEIVSYAAKGEEGKSLAVLREGVIKTEAPERLREDCRLSTEPASNGLAHGNKDVILTQPSSAFGPHQQ  270
Buffalo_SOX-30  KAEGSGPLNSRRGEEKKGKLEAEEIVSYAAKGEEGKSLAVLREGVIKTEVPERLREDCRLSTEPASNGLAHGSKDVILTQPSSAFGPHQQ  270
Goat_SOX-30     KAEGSGPLNSRRGDEKKGKLEAEEIVSYAAKGEEGKSLAALREGVIKTEAPERLREDCRLSTEPASNGLAHGSKDVILTQPSTAFGPHQQ  270
Sheep_SOX-30    KGEGSGPLNSRRGDEKKGKLEAEEIVSYSAKGEEGKSLAALREGGIKTEAPERLREDCRLSTEPASNGLAHGSKDVILTQPSTAFGPHQQ  270

Cow_SOX-30      DLRIPLTLHTVPPGARIQFQGPPPSELIRLTKVPLTPVPIKMQSLLEPSVKIETKDVPLTVLPSDAGIPDTPFSKDRNGHVKRPMNAFMV  360
Buffalo_SOX-30  DLRIPLTLHTVPPGARIQFQGPPPSELIRLTKVPLTPVPIKMQSLLEPSVKIETKDVPLTVLPSDAGIPDTPFSKDRNGHVKRPMNAFMV  360
Goat_SOX-30     DLRIPLTLHTVPPGARIQFQGPPPSELIRLTKVPLTPVPIKMQSLLEPSVKIETKDVPLTVLPSDAGIPDTPFSKDRNGHVKRPMNAFMV  360
Sheep_SOX-30    DLRIPLTLHTVPPGARIQFQGPPPSELIRLTKVPLTPVPIKMQSLLEPSVKIETKDVPLTVLPSDAGIPDTPFSKDRNGHVKRPMNAFMV  360

Cow_SOX-30      WARIHRPALAKANPAANNAEISVQLGLEWNKLSEEQKKPYYDEAQKIKEKHREEFPGWVYQPRPGKRKRFPLSVSSVFSGTTQNIISTNP  450
Buffalo_SOX-30  WARIHRPALAKANPAANNAEISVQLGLEWNKLSEEQKKPYYDEAQKIKEKHREEFPGWVYQPRPGKRKRFPLSVSSVFSGTTQNIISTNP  450
Goat_SOX-30     WARIHRPALAKANPAANNAEISVQLGLEWNKLSEEQKKPYYDEAQKIKEKHREEFPGWVYQPRPGKRKRFPLSVSNVFSGTTQNIISTNP  450
Sheep_SOX-30    WARIHRPALAKANPAANNAEISVQLGLEWNKLSEEQKKPYYDEAQKIKEKHREEFPGWVYQPRPGKRKRFPLSVSNVFSGTTQNIISTNP  450

Cow_SOX-30      -TIYPYRSPTYSVVIPSLQNAITHPVGESPPTIQLPTPAVQRPSPITLFQPSVSSTAQVAVQAPSLPLRPALPPQRFAGPSQADTHQLHS  539
Buffalo_SOX-30  -TIYPYRSPTYSVVIPSLQNAITHPVGESPPTIQLPTPAVQRPSPITLFQPSVSSTAQVAVQAPSLPLRPALPPQRFAGPSQADTHQLHS  539
Goat_SOX-30     TTIYPYRSPTYSVVIPSLQNTITHPVGESPPTIQLPTPAVQRPSPITLFQPSVSSTAQVPVQAPSLPLRPALPPQRFAGPSQTDTHRLHS  540
Sheep_SOX-30    TTIYPYRSPTYSVVIPSLQNTITHPVGESPPTIQLPTPAVQRPSPITLFQPSVSSTAQVPVQAPSLPLRPALPPQRFAGPSQADTHRLHS  540

Cow_SOX-30      GVSRSVKRPTPVSLESTNRIPTSASPAHSRFATSTIQPPKEYPSVSTCPRSTPISQAPPIPHSHIYQPSPLGHPAALFGTPPRFSFHHPY  629
Buffalo_SOX-30  GVNRSVKRPTPVSLENTNRIPTSASPAHSRFATSTIQPPKEYPSVSTCPRNTPISQAPPIPHSHVFQPPPLGHPAALFGTPPRFSFHHPY  629
Goat_SOX-30     GVNRSVKRPTPVSLESTNRIPTSASPAHSRFSTSTIQPPKEYPSVSTCPRSTPISQAPPIPHSHVYQPPPLGHPAALFGTPPRFSFHHPY  630
Sheep_SOX-30    GVNRSVKRPTPVSLESTNRIPTSASPAHSRFSTSTIQPPKEYPSVSTCPRSTPISQAPPIPHSHVYQPPPLGHPAALFGTPPRFSFHHPY  630

Cow_SOX-30      FLPGPHYFPSSTCPYSRPPFGYGNFPSSMPECLGYYEDRYQKHEAMFSALNRDYPFRDYPDERAHNEDSRSCENMDGTSYYNSHCHSGEE  719
Buffalo_SOX-30  FLPGPHYFPSSTCPYSRPPFGYGNFPSSMPECLGYYEDRYQKHEAMFSALNRDYPFRDYPDERAHNDDSRSCENMDGTSYYNSHCHSGEE  719
Goat_SOX-30     FLPGPHYFPSSTCPYSRPPFGYGNFPSSMPECLGYYEDRYQKHEAMFSALNRDYPFRDYPDERAHNEDSRSCENMDGTSYYNSHSHSGEE  720
Sheep_SOX-30    FLPGPHYFPSSTCPYSRPPFGYGNFPSSMPECLGYYEDRYQKHEAMFSALNRDYPFRDYPDERAHNEDSRSCENMDGTSYYNSHSHSGEE  720

Cow_SOX-30      YLNPIPQLDIGALENVFTAPTSTPSSIQQVNVTDSDEEEEEKVLRNL  766
Buffalo_SOX-30  YLNPMPQLDIGALENVFTAPTSTPSSIQQVNVTDSDEEEEEKVLRNL  766
Goat_SOX-30     YLNPMPQLDIGALENVFTAPTSTPSSIQQVNVTDSDEEEEEKVLRNL  767
Sheep_SOX-30    YLNPMPQLDIGALENVFTAPTSTPSSIQQVNVTDSDEEEEEKVLRNL  767
```

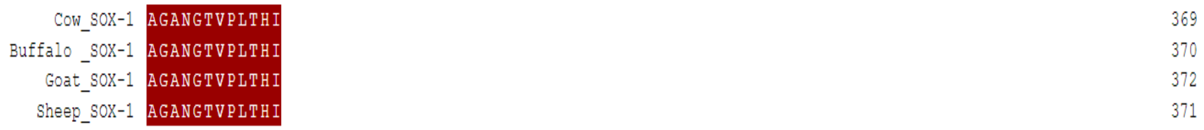

Figure S19. Comparative amino acid analysis of Sox30 gene in cattle, buffalo, sheep, and goat

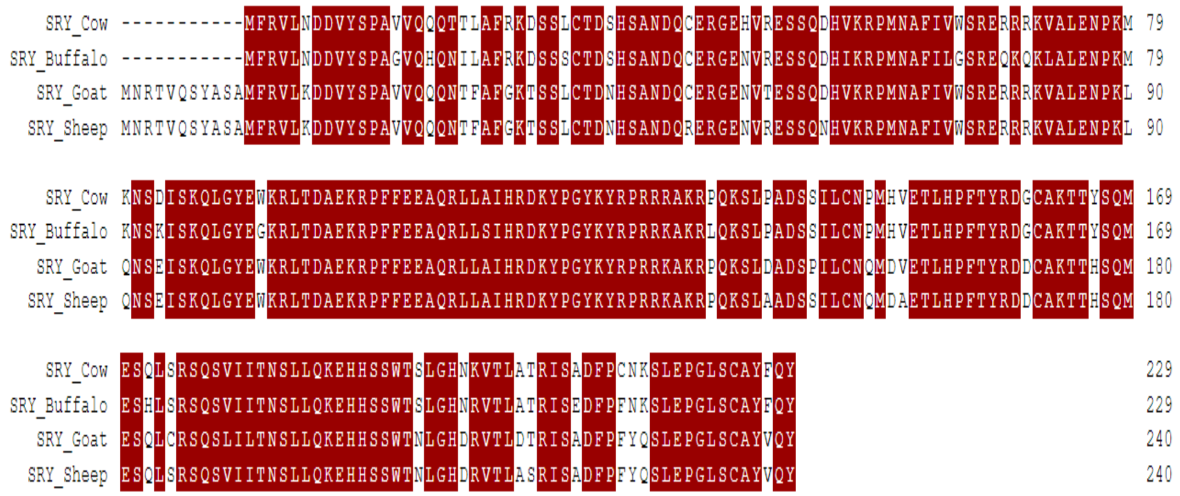

Figure S20. Comparative amino acid analysis of Sry gene in cattle, buffalo, sheep, and goat

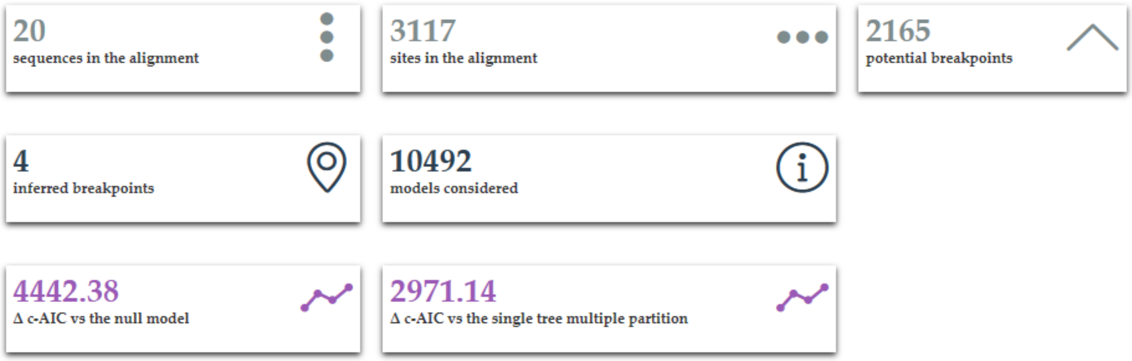

Figure S21. Analysis of GARD

Table S1: Buffalo and cattle Sox gene family with chromosome number and location

Gene	Buffalo		Cattle	
	Chromosome	Position	Chromosome	Position
SOX1	13	1124390…1127795	12	86018990..86022110
SOX2	1	130731784…130734338	1	85302293..85303771
SOX3	X	118463501…118465239	Unplaced Scaffold	4426849..4429001
SOX4	2	15869169…15873430	23	36517935..36522538
SOX5	4	33262643…34419596	5	85456814..86631212
SOX6	16	48888897…49605774	15	35834420..36548505
SOX7	3	71168529…71174671	8	8558547..8565620
SOX8	24	41398973…41403986	25	788544..793550
SOX9	3	4493353…4498743	19	58918901..58922699
SOX10	4	10196777…10207847	5	109757715..109768623
SOX11	12	90353529…90362307	11	90946223..90955282
SOX12	14	22918829…22921779	13	60689766..60694687
SOX13	5	79338823…79383069	16	1867662..1912526
SOX14	1	176274075…176276138	1	131371915..131372637
SOX15	3	35785580…35787156	19	27319235..27321042
SOX17	15	59969970…59975876	14	22229926..22231726
SOX18	14	29832918…29834810	13	53823431..53825287
SOX21	13	20645934…20650444	12	69200712..69204003
SOX30	9	41564987…41608436	7	69215442..69297855
Sry	Y	150…839	Y	42225210..42225899

Table S2. Functional effect of mutations in Sox genes in buffalo

	Mutation	Polyphen2	Mupro	Provean	I-Mutant	Phd-Snp	SIFT	SNAP^2	Predict SNP	Meta SNP	SNAP	
SOX-1												
	**A223P**	BENIGN	DECREASE	Neutral	Increase	Neutral	Tolerated	Effect	Neutral	Neutral	NA	Synonymous
**SOX-4**												
	**T225A**	UNKNOWN	DECREASE	Neutral	Decrease	Neutral	Tolerated	Neutral	Neutral	Neutral	NA	Synonymous
SOX-5												
	**A84T**	BENIGN	DECREASE	Neutral	Decrease	Neutral	Tolerated	Neutral	Neutral	Neutral	NA	Synonymous
	**P362S**	BENIGN	DECREASE	Deleterious	Decrease	Neutral	Not Tolerated	Effect	Neutral	Neutral	NA	Non Synonymous
SOX-6												
	**L2P**	UNKNOWN	DECREASE	Neutral	Decrease	Neutral	Not Tolerated	Neutral	Neutral	Neutral	NA	Non Synonymous
	**E45D**	BENIGN	INCREASE	Neutral	Decrease	Neutral	Tolerated	Effect	Neutral	Neutral	NA	Synonymous
	**S66P**	BENIGN	INCREASE	Neutral	Increase	Neutral	Tolerated	Effect	Neutral	Neutral	NA	Synonymous
	**I138M**	BENIGN	DECREASE	Neutral	Decrease	Neutral	Tolerated	Neutral	Neutral	Neutral	NA	Synonymous
	**S476P**	BENIGN	DECREASE	Neutral	Increase	Neutral	Tolerated	Neutral	Neutral	Neutral	NA	Synonymous
SOX-7												
	**R168S**	BENIGN	DECREASE	Neutral	Decrease	Neutral	Tolerated	Effect	Neutral	Neutral	Neutral	Synonymous
	**S264G**	BENIGN	DECREASE	Neutral	Decrease	Neutral	Tolerated	Neutral	Neutral	Neutral	Neutral	Synonymous
	**P276S**	BENIGN	DECREASE	Neutral	Decrease	Neutral	Tolerated	Effect	Neutral	Neutral	Neutral	Synonymous
	**A352G**	BENIGN	DECREASE	Neutral	Decrease	Neutral	Tolerated	Neutral	Neutral	Neutral	Neutral	Synonymous
SOX-8												
	**R42H**	POSSIBLY DAMAGING	INCREASE	Neutral	Decrease	Neutral	Not Tolerated	Effect	Neutral	Neutral	NA	Synonymous
	**T43A**	BENIGN	DECREASE	Neutral	Decrease	Neutral	Not Tolerated	Effect	Neutral	Neutral	NA	Synonymous
	**S270G**	BENIGN	DECREASE	Neutral	Decrease	Neutral	Tolerated	Neutral	Neutral	Neutral	NA	Synonymous
	**T382A**	BENIGN	INCREASE	Neutral	Decrease	Neutral	Tolerated	Neutral	Neutral	Neutral	NA	Synonymous
	**S391G**	PROBABLY DAMAGING	DECREASE	Neutral	Decrease	Neutral	Tolerated	Effect	Neutral	Neutral	NA	Synonymous
	**Y443F**	BENIGN	INCREASE	Neutral	Decrease	Disease	Not Tolerated	Effect	Deleterious	Neutral	NA	Non-Synonymous
SOX-9												
	**A372P**	UNKNOWN	DECREASE	Neutral	Increase	Neutral	Tolerated	Effect	Neutral	Neutral	NA	Synonymous
	**P374Q**	UNKNOWN	DECREASE	Neutral	Increase	Disease	Tolerated	Neutral	Neutral	Neutral	NA	Synonymous
SOX-11												
	**G351S**	UNKNOWN	DECREASE	Neutral	Increase	Neutral	Tolerated	Neutral	Neutral	Neutral	NA	Synonymous
SOX-13												

	**G5R**	UNKNOWN	INCREASE	Neutral	Increase	Neutral	Not Tolerated	Effect	Deleterious	Neutral	Disease	Non-Synonymous
	**F20V**	UNKNOWN	DECREASE	Neutral	Decrease	Neutral	Not Tolerated	Effect	Deleterious	Disease	Disease	Non-Synonymous
	**F39L**	UNKNOWN	DECREASE	Neutral	Decrease	Neutral	Not Tolerated	Effect	Neutral	Disease	Disease	Non-Synonymous
	**T83A**	BENIGN	DECREASE	Neutral	Decrease	Neutral	Tolerated	Neutral	Neutral	Neutral	Disease	Synonymous
	**T95I**	BENIGN	INCREASE	Neutral	Increase	Neutral	Not Tolerated	Effect	Neutral	Neutral	Disease	Synonymous
	**T106A**	BENIGN	INCREASE	Neutral	Decrease	Neutral	Tolerated	Neutral	Neutral	Neutral	Neutral	Synonymous
	**S116L**	BENIGN	INCREASE	Neutral	Decrease	Neutral	Not Tolerated	Neutral	Neutral	Neutral	Disease	Synonymous
	**G166D**	BENIGN	DECREASE	Neutral	Decrease	Neutral	Tolerated	Neutral	Neutral	Neutral	Neutral	Synonymous
	**A315T**	BENIGN	DECREASE	Neutral	Decrease	Neutral	Tolerated	Neutral	Neutral	Neutral	Neutral	Synonymous
	**G610S**	BENIGN	DECREASE	Neutral	Decrease	Neutral	Tolerated	Neutral	Neutral	Neutral	Neutral	Synonymous
	**V642M**	BENIGN	DECREASE	Neutral	Decrease	Neutral	Tolerated	Effect	Neutral	Neutral	Neutral	Synonymous
	SOX-15											
	**D15E**	BENIGN	DECREASE	Neutral	Increase	Neutral	Tolerated	Neutral	Neutral	Neutral	Neutral	Synonymous
	**S124T**	BENIGN	INCREASE	Neutral	Decrease	Neutral	Tolerated	Neutral	Neutral	Neutral	Disease	Synonymous
	**T205P**	DAMAGING	DECREASE	Neutral	Decrease	Neutral	Not Tolerated	Effect	Neutral	Neutral	NA	Non-Synonymous
	**P211L**	BENIGN	DECREASE	Neutral	Decrease	Neutral	Tolerated	Effect	Neutral	Neutral	Neutral	Synonymous
	SOX-17											
	**A52G**	BENIGN	DECREASE	Neutral	Decrease	Neutral	Tolerated	Neutral	Neutral	Neutral	Neutral	Synonymous
	**P374S**	UNKNOWN	DECREASE	Neutral	Decrease	Neutral	Tolerated	Neutral	Neutral	Neutral	Neutral	Synonymous
	SOX-18											
	**A314V**	BENIGN	DECREASE	Neutral	Increase	Neutral	Tolerated	Neutral	Neutral	Neutral	NA	Synonymous
	**S363T**	PROBABLY DAMAGING	INCREASE	Neutral	Increase	Neutral	Tolerated	Neutral	Neutral	Neutral	NA	Synonymous
	SOX-21											
	**A119T**	BENIGN	INCREASE	Neutral	Decreases	Neutral	Not Tolerated	Neutral	Neutral	Neutral	NA	Synonymous
	SOX-30											
	**P21S**	UNKNOWN	DECREASE	Neutral	Decrease	Neutral	Tolerated	Neutral	Neutral	Neutral	Neutral	Synonymous
	**P28A**	UNKNOWN	DECREASE	Neutral	Decrease	Neutral	Not Tolerated	Effect	Neutral	Neutral	Neutral	Non-Synonymous
	**A42V**	POSSIBLY DAMAGING	INCREASE	Neutral	Increase	Neutral	Not Tolerated	Neutral	Deleterious	Neutral	Neutral	Synonymous
	**C53W**	PROBABLY DAMAGING	INCREASE	Neutral	Increase	Neutral	Not Tolerated	Effect	Deleterious	Disease	Disease	Non-Synonymous
	**S103G**	BENIGN	DECREASE	Neutral	Decrease	Neutral	Not Tolerated	Neutral	Neutral	Neutral	Neutral	Synonymous

	**P118S**	PROBABLY DAMAGING	DECREASE	Neutral	Decrease	Neutral	Not Tolerated	Neutral	Neutral	Neutral	Neutral	Synonymous
	**T179R**	BENIGN	DECREASE	Neutral	Increase	Neutral	Not Tolerated	Effect	Deleterious	Neutral	Disease	Non-Synonymous
	**A230V**	BENIGN	INCREASE	Neutral	Decrease	Neutral	Not Tolerated	Neutral	Neutral	Neutral	Neutral	Synonymous
	**N253S**	BENIGN	DECREASE	Neutral	Decrease	Neutral	Tolerated	Neutral	Neutral	Neutral	Neutral	Synonymous
	**S542N**	BENIGN	INCREASE	Neutral	Increase	Neutral	Not Tolerated	Neutral	Neutral	Neutral	Disease	Synonymous
	**S555N**	BENIGN	DECREASE	Neutral	Decrease	Neutral	Not Tolerated	Effect	Neutral	Neutral	Neutral	Synonymous
	**S590N**	BENIGN	DECREASE	Neutral	Decrease	Neutral	Not Tolerated	Effect	Neutral	Neutral	Disease	Synonymous
	**I604V**	BENIGN	DECREASE	Neutral	Increase	Neutral	Tolerated	Neutral	Neutral	Neutral	Neutral	Synonymous
	**Y605F**	PROBABLY DAMAGING	DECREASE	Neutral	Increase	Neutral	Not Tolerated	Effect	Deleterious	Disease	Disease	Non-Synonymous
	**S608P**	BENIGN	INCREASE	Neutral	Increase	Neutral	Tolerated	Neutral	Neutral	Neutral	Neutral	Synonymous
	**E696D**	PROBABLY DAMAGING	DECREASE	Neutral	Decrease	Neutral	Not Tolerated	Effect	Deleterious	Neutral	Neutral	Non-Synonymous
	**I724M**	BENIGN	DECREASE	Neutral	Decrease	Neutral	Tolerated	Neutral	Neutral	Neutral	Neutral	Synonymous
	SRY											
	**V14G**	BENIGN	DECREASE	Neutral	Decrease	Neutral	Tolerated	Neutral	Neutral	Neutral	NA	Synonymous
	**Q17H**	BENIGN	DECREASE	Neutral	Decrease	Neutral	Tolerated	Effect	Neutral	Neutral	NA	Synonymous
	**T19N**	BENIGN	INCREASE	Neutral	Decrease	Neutral	Tolerated	Neutral	Neutral	Neutral	NA	Synonymous
	**L29S**	BENIGN	DECREASE	Neutral	Decrease	Neutral	Not Tolerated	Effect	Neutral	Neutral	NA	Synonymous
	**H45N**	BENIGN	INCREASE	Neutral	Decrease	Neutral	Tolerated	Neutral	Neutral	Neutral	NA	Synonymous
	**V54I**	BENIGN	DECREASE	Neutral	Increase	Neutral	Tolerated	Neutral	Neutral	Neutral	NA	Synonymous
	**V63L**	BENIGN	DECREASE	Deleterious	Decrease	Disease	Not Tolerated	Effect	Deleterious	Disease	NA	Non-Synonymous
	**W64G**	BENIGN	DECREASE	Deleterious	Decrease	Disease	Not Tolerated	Effect	Deleterious	Disease	NA	Non-Synonymous
	**R68Q**	BENIGN	DECREASE	Neutral	Decrease	Neutral	Tolerated	Neutral	Neutral	Neutral	NA	Synonymous
	**R69K**	BENIGN	DECREASE	Deleterious	Decrease	Disease	Not Tolerated	Effect	Deleterious	Disease	NA	Non-Synonymous
	**R70Q**	BENIGN	DECREASE	Deleterious	Decrease	Disease	Not Tolerated	Effect	Deleterious	Disease	NA	Non-Synonymous
	**V72L**	BENIGN	INCREASE	Neutral	Increase	Neutral	Not Tolerated	Neutral	Neutral	Neutral	NA	Synonymous
	**D83K**	BENIGN	DECREASE	Neutral	Decrease	Disease	Not Tolerated	Effect	Deleterious	Disease	NA	Non-Synonymous

	**W92G**	BENIGN	DECREASE	Deleterious	Decrease	Disease	Not Tolerated	Effect	Deleterious	Disease	NA	Non-Synonymous
	**A112S**	BENIGN	DECREASE	Neutral	Decrease	Disease	Not Tolerated	Effect	Neutral	Disease	NA	Non-Synonymous
	**R128K**	BENIGN	DECREASE	Neutral	Decrease	Neutral	Tolerated	Neutral	Neutral	Neutral	NA	Synonymous
	**P132L**	BENIGN	INCREASE	Neutral	Decrease	Neutral	Tolerated	Neutral	Neutral	Neutral	NA	Synonymous
	**Q172H**	BENIGN	DECREASE	Deleterious	Decrease	Disease	Not Tolerated	Effect	Neutral	Neutral	NA	Non-Synonymous
	**K201R**	BENIGN	DECREASE	Neutral	Increase	Neutral	Tolerated	Effect	Neutral	Neutral	NA	Synonymous
	**A210E**	BENIGN	DECREASE	Neutral	Decrease	Neutral	Not Tolerated	Effect	Neutral	Neutral	NA	Synonymous
	**C214F**	BENIGN	DECREASE	Neutral	Increase	Neutral	Tolerated	Effect	Neutral	Neutral	NA	Synonymous
